# Evidence review and considerations for use of first line genome sequencing to diagnose rare genetic disorders

**DOI:** 10.1038/s41525-024-00396-x

**Published:** 2024-02-26

**Authors:** Kristen M. Wigby, Deanna Brockman, Gregory Costain, Caitlin Hale, Stacie L. Taylor, John Belmont, David Bick, David Dimmock, Susan Fernbach, John Greally, Vaidehi Jobanputra, Shashikant Kulkarni, Elizabeth Spiteri, Ryan J. Taft

**Affiliations:** 1 University of California, Davis, CA USA; 2 Rady Children’s Institute for Genomic Medicine, San Diego, CA USA; 3 Massachusetts General Hospital, Boston, MA USA; 4 The Hospital for Sick Children, Toronto, CA USA; 5 Stanford Healthcare, Palo Alto, CA USA; 6 Illumina Inc, San Diego, CA USA; 7 Genetics & Genomics Services Inc, Houston, TX USA; 8 Genomics England Ltd, London, UK; 9 Baylor College of Medicine, Houston, TX USA; 10 Albert Einstein College of Medicine, Bronx, NY USA; 11 New York Genome Center, New York, NY USA

**Keywords:** Genetic testing, Medical genomics, Genetic variation, DNA sequencing

## Abstract

Early use of genome sequencing (GS) in the diagnostic odyssey can reduce suffering and improve care, but questions remain about which patient populations are most amenable to GS as a first-line diagnostic test. To address this, the Medical Genome Initiative conducted a literature review to identify appropriate clinical indications for GS. Studies published from January 2011 to August 2022 that reported on the diagnostic yield (DY) or clinical utility of GS were included. An exploratory meta-analysis using a random effects model evaluated DY based on cohort size and diagnosed cases per cohort. Seventy-one studies met inclusion criteria, comprising over 13,000 patients who received GS in one of the following settings: hospitalized pediatric patients, pediatric outpatients, adult outpatients, or mixed. GS was the first-line test in 38% (27/71). The unweighted mean DY of first-line GS was 45% (12–73%), 33% (6–86%) in cohorts with prior genetic testing, and 33% (9–60%) in exome-negative cohorts. Clinical utility was reported in 81% of first-line GS studies in hospitalized pediatric patients. Changes in management varied by cohort and underlying molecular diagnosis (24–100%). To develop evidence-informed points to consider, the quality of all 71 studies was assessed using modified American College of Radiology (ACR) criteria, with five core points to consider developed, including recommendations for use of GS in the N/PICU, in lieu of sequential testing and when disorders with substantial allelic heterogeneity are suspected. Future large and controlled studies in the pediatric and adult populations may support further refinement of these recommendations.

## Introduction

Short-read whole genome sequencing (referred to herein as genome sequencing or GS) has accelerated knowledge of genetic contributions to human disease. Since the completion of the Human Genome Project in 2003, GS has evolved from a powerful research technology to a clinically validated assay that can robustly and routinely deliver diagnostic findings for individuals with rare genetic disorders^1,2^. These conditions, which have diverse presentations inclusive of single and multi-system organ involvement, are linked by pathogenic DNA variations that span from single nucleotide changes to complex chromosomal rearrangements. Before the introduction of untargeted diagnostic sequencing approaches such as exome sequencing (ES) and GS, a patient’s presenting features (also referred to as phenotype, see Table 1 for examples) were used to guide the selection of the appropriate targeted genetic test(s) to confirm a diagnosis. GS testing provides the opportunity for near-comprehensive diagnostic evaluation across thousands of genetic disorders in parallel for patients with a clinical suspicion of a rare genetic disorder, including those with non-specific presentations.

**Table 1:** Signs and symptoms of genetic disorders*

• Clinical features not explained by acquired causes and known to be associated with multiple genetic disorders
• e.g., intellectual disability, microcephaly, hypertrophic cardiomyopathy
• Family history consistent with a Mendelian pattern of inheritance and/or mitochondrial inheritance
• Presentation of an adult-onset disorder at a younger age than expected for the disorder
• Atypical course for a disorder including unusually severe course or prolonged duration
• Lack of response or atypical response to standard therapy for an acquired disorder
• Rare and significant clinical or laboratory findings
• e.g., congenital structural anomalies, unusual histologic findings on biopsy, specific, rare, and clinically significant deviations in laboratory results

*Adapted with permission from Bick et al., J Med Genet 2019^40^.

Historically, identifying a rare genetic disorder was often challenging due either to the nature of the phenotype or to the underlying molecular mechanism(s) of the disease. For example, a phenotype such as severe global developmental delay may be highly suggestive of a genetic disorder but not specific to a single recognizable condition. Phenotypes may be associated with multiple genes (locus heterogeneity) and types of genetic variation (allelic heterogeneity), each of which could require a targeted genetic test to identify. Also, complex or “blended” phenotypes due to the presence of multiple distinct genetic disorders within the same individual, have been estimated to occur in 2–4% of individuals receiving genomic sequencing^3–5^.

Most clinically available genetic tests (e.g., karyotype, chromosomal microarray analysis, gene panels) are restricted in scope and assess limited genetic conditions, loci, and/or classes of genetic variation. Exome sequencing, which encompasses sequencing of exons or protein-coding portions of the ~20,000 genes in the nuclear genome, is considered by many a genome-wide test^6^. In contrast, GS assesses almost the entire genome of an individual, including non-coding and deep intronic regions not included in ES. Furthermore, while ES can detect some copy number variations (CNVs), the uniform sequencing and coverage of the genome by GS optimizes CNV variant detection and enables the detection of copy-neutral structural variation^7^. GS can also be used to identify certain repeat expansion disorders, which are inaccessible with ES and panel-based genetic tests. GS overcomes the diagnostic challenges of targeted tests by simultaneously evaluating multiple genetic loci and multiple variant types^8^, which enables discrimination of phenotypically similar conditions, including those not considered by the clinician in the differential diagnosis. Genome-wide analysis removes the barrier to rare disease diagnosis posed by more targeted conventional genetic tests.

As genetic tests are increasingly deployed in healthcare, an important question remains: when, how, and for whom should GS be offered as opposed to other available genetic tests? This question has been challenging to answer as various participants in clinical genetic testing, including clinicians, healthcare systems, and payers, apply differing criteria in their selection of patients to receive genetic testing^4,9–12^. For many with rare genetic disorders, the diagnostic evaluation is a multi-year process involving multiple tests and evaluations with specialists^13^. Social determinants of health, including race and ethnicity, socioeconomic status, and geographical location create additional barriers to access a diagnostic evaluation for rare genetic disorders for some individuals^9^. Presently, there are more than 700 childhood-onset treatable genetic disorders where the timely diagnosis may avoid the morbidity associated with a specific condition and, in some cases, allow for life-saving interventions^10^. GS is poised to address this unmet diagnostic need. While sequencing costs are projected to drop significantly, the likely lag in reimbursement for clinical GS testing necessitates guidance to identify patients most likely to benefit. While published guidelines and health technology assessments have sought to identify patient populations that would most benefit from ES or GS^4,11,12,14,15^, they do not provide practice recommendations for GS use as a first-line diagnostic test for rare genetic disorders. Further, clinical trials cannot evaluate every possible diagnostic scenario and expert opinion is needed to fill these evidentiary gaps.

To address these challenges, an expert review panel was convened by the Medical Genome Initiative, a consortium of leading clinical GS testing institutions^16^. This included a focused literature review to examine the contexts in which GS has been applied as a first-line genetic test. Secondly, we conducted an exploratory meta-analysis using a random effects model of proportions to generate a point estimate for diagnostic yield that accounted for cohort size and the number of diagnosed cases per cohort. Informed by our findings and expert opinion from the working group, we developed five recommendations for GS testing patient identification.

## Results

### Search results and study characteristics

**Fig. 1:**
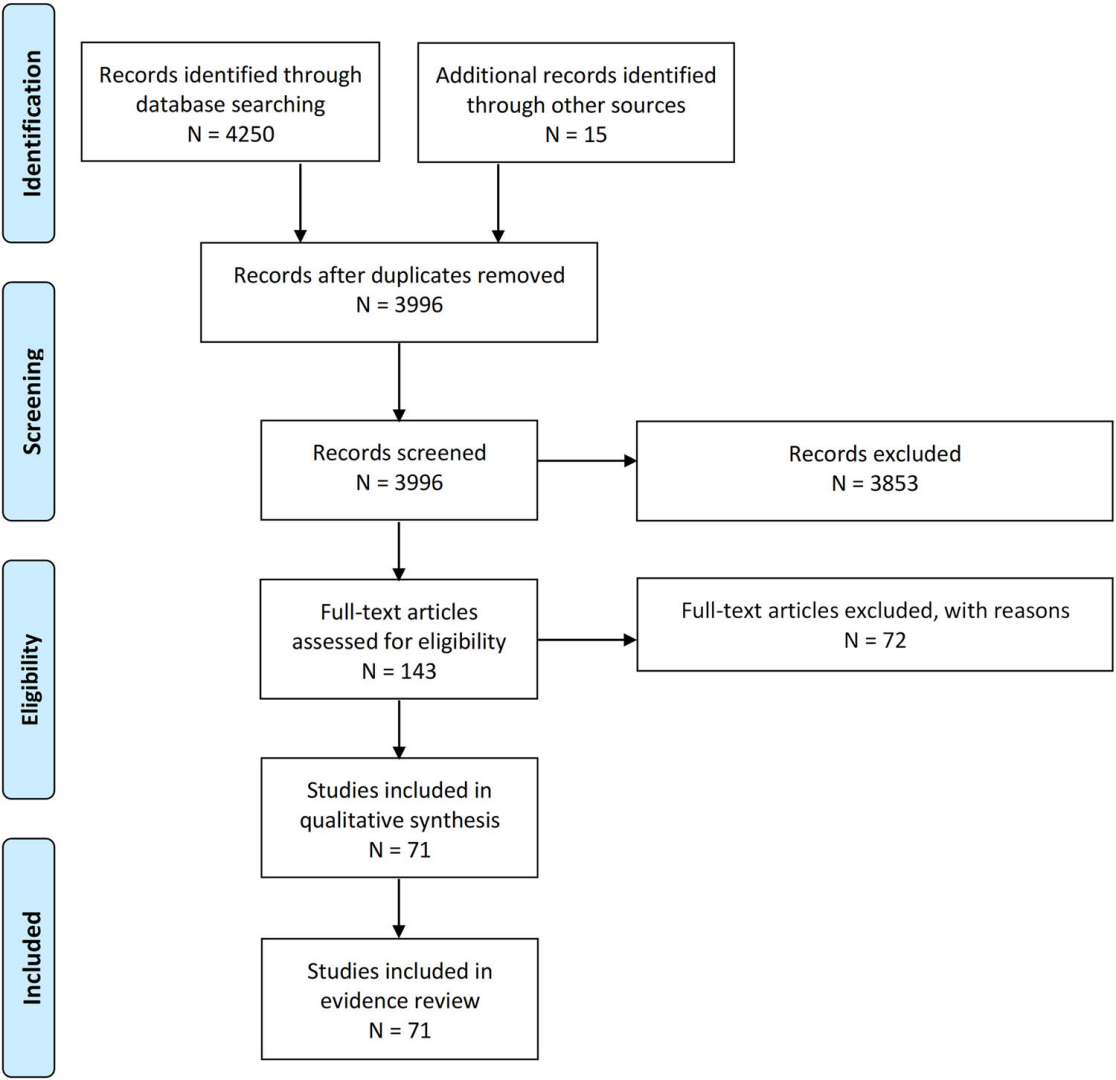
PRISMA study selection flowchart. *Reasons for exclusion included inappropriate publication type or study design (e.g., case reports), lack of primary outcome measures, and secondary publications.

Database searches of PubMed and Dimensions yielded 4250 records plus fourteen additional references that were identified through other sources (e.g., manuscript bibliographies and hand searching of journals). A total of 269 duplicates were removed. After title and abstract screening were performed on the remaining 3995 references, 3853, records were excluded based on the inclusion and exclusion criteria (see Methods). One hundred forty-three articles underwent additional full-text screening for eligibility and 72 were excluded, including removal of case reports (*n* = 32). Two clinical trial cohorts (NSIGHT1 and NSIGHT2) were reported in more than one publication and were “linked” to avoid overestimating the number of unique dataset publications. Thus, a total of 71 studies were included in the evidence review (Fig. 1).

### Study characteristics

The 71 studies meeting inclusion criteria were published between January 2014 to August 2022. Supplementary Tables 1–4 summarize the included studies, which are grouped into one of four categories according to healthcare setting: pediatric hospital cohorts, pediatric ambulatory cohorts, adult cohorts, and mixed cohorts.

**Fig. 2:**
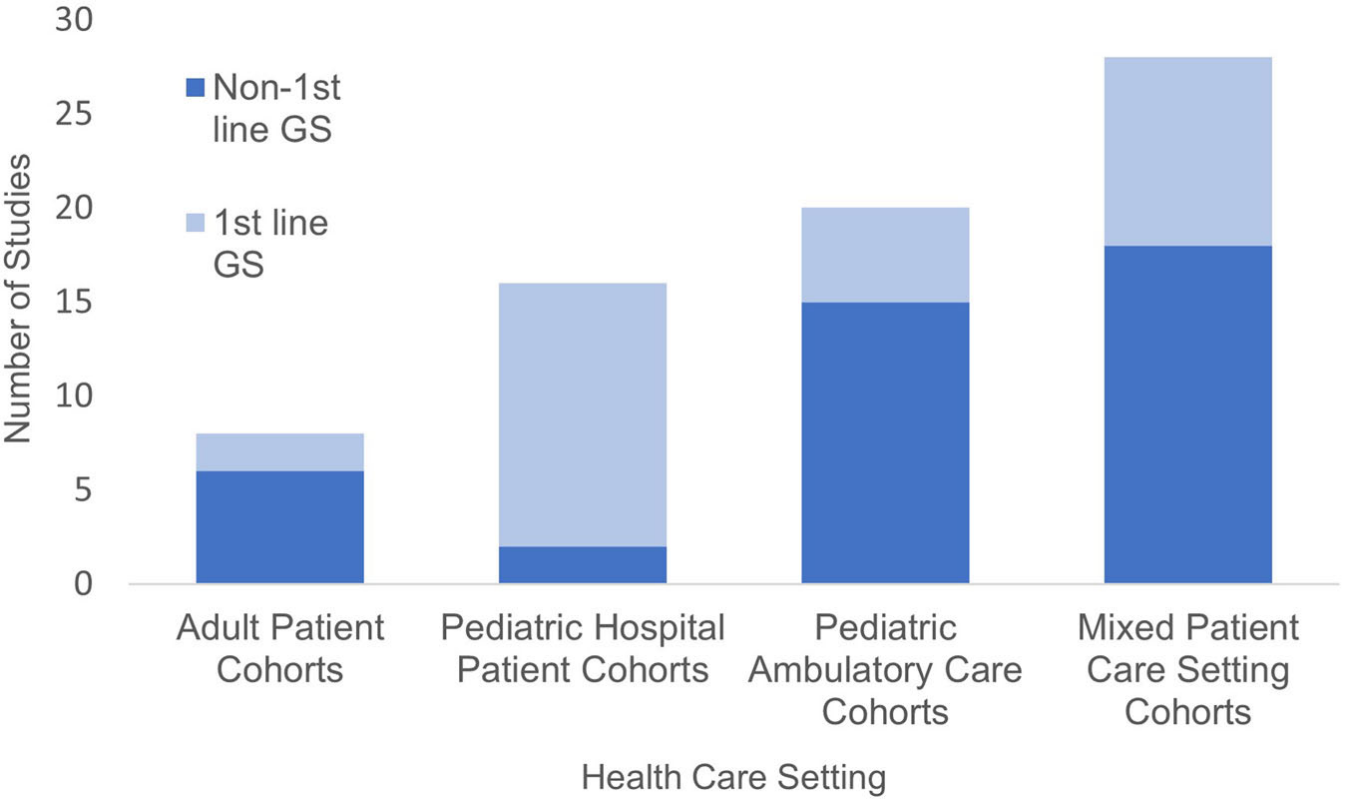
Care setting with a breakdown of first-line GS. Number of studies by healthcare setting, including studies where GS was the first-line genetic test (dark blue) and where GS was not the first-line genetic test (light blue).

The number of GS studies published per year gradually increased with 10 or more studies published from 2018 to 2021 (range 10–13). One or more studies were conducted in ten different countries spanning four continents (including Asia, Europe, North America, and Oceania). Forty-four percent (31/71) of studies were conducted by institutions not affiliated with the Medical Genome Initiative. Studies varied in size ranging from cohorts up to 20 patients (9/71) to studies including more than 100 patients (24/71). Combined, these studies performed GS on over 13,000 patients across diverse care settings (Fig. 2).

Studies differed with respect to analysis strategy. Thirty studies (42%, 30/71) employed a panel-based initial approach based on a targeted set of established disease genes associated with the primary phenotype and variable sequential use of broader secondary gene panels or reflex to an untargeted approach. The remaining 41 studies used a phenotype-informed untargeted analysis. Phenotypic features were frequently used in the untargeted approach to provide additional prioritization of genes, but these studies did not limit the analysis to a pre-specified gene list. Additional gene-discovery analyses of candidate genes, using various methods, including investigations of rare, predicted deleterious coding variants in genes without an established human disease association, were reported in 38 studies. Analyses of non-coding variants (e.g., deep intronic, intergenic, untranslated region (UTR) variants) were conducted in 35 studies. Many non-coding analyses focused on non-coding variants present in relevant, established genes related to the phenotype or a single type of non-coding variation (most frequently deep intronic variation). A subset further limited non-coding analysis to only previously known pathogenic variants included in curated datasets (e.g., only UTR promoter or intronic variants reported in Human Gene Mutation Database (HGMD) or Clinvar)^17–19^. Other studies focused on non-coding variant analysis on specific scenarios, such as the detection of a single deleterious coding variant associated with an autosomal recessive disease gene.

Twenty-two studies used proband-only analysis, 27 studies analyzed parent-proband trios, and 22 studies included a mixture (e.g., trios with an option to include samples with only one parent available, analysis of additional affected siblings or other family members).

Fifty-nine studies applied the 2015 ACMG guidelines for variant interpretation and classification^20^ and the remainder of studies applied earlier guidelines^21^ or other systematic approaches^22^. Fifty-eight studies also described variants classified as variants of uncertain significance (VUS) in established disease genes. The overwhelming majority of studies reported classified or asserted pathogenic single nucleotide variants (SNVs) in known disease-causing protein-coding genes (97%, 69/71) associated with some or all of the phenotypic features. The two studies that did not report SNVs focused the analysis on copy number variants and other structural variations in a cohort of apparently balanced chromosomal anomalies^23^ or a cohort of undiagnosed rare disease^24^. Other commonly reported variants included indels (89%, 63/71) and CNVs (73%, 52/71). Less commonly reported variant types included mitochondrial DNA (mt-DNA; 20%, 14/71) variants, non-CNV structural variants (34%, 24/71), mosaic variants (11%, 8/71), and short tandem repeats including repeat expansions (14%, 10/71). Overall, the range of variant types reported increased from 2018 to 2022, with more studies reporting mt-DNA variants and non-CNV structural variants. Studies differed in the detail regarding results returned to clinicians and/or study participants which made estimates of reporting candidate genes or non-coding variants challenging, although some studies conducted such analyses. Secondary findings were reported in only 27% (19/71) of the included studies.

Seventeen studies reported turn-around time which ranged from three to 73 days. Of these 17 studies, six (35%) reported a turn-around time of 10 days or less (see Table 2).

**Table 2:** Turn-around time

Study	Median TAT provisional in days (Range)	Median TAT final in days (Range)	Comments
Bowling et al., 2022^41^		73 (6–129)	
Dimmock et al., 2021^27^		3 (NR)	
Farnaes et al., 2018^42^	7 (3–12)	23 (5–69)	
French et al., 2019^43^		<35 (21–175)	
Kingsmore et al., 2019^44^	11 (3.3-49.1)		Ultra-rapid cases 4.6 (1.1–14)
Mestek-Boukhibar et al., 2018^45^		11.5 (4–42)	
Palmquist et al., 2022^46^	4 (1–16)	11 (4–27)	
Petrikin et al., 2018^47^		14 (8–35)	
Sanford et al., 2019^48^		13.6 (1-56)	
Sweeney et al., 2021^49^		16.5 (4–34)	
Van Diemen et al., 2018^50^		12 (5–23)	
Wang et al., 2021^51^		3.9 (3-4)	
Willig et al., 2015^52^		23 (5-912)	
Wu et al., 2021^53^		7 (5–20)	

The majority of studies were conducted in mixed patient care settings (39%, 28/71), which included a combination of ambulatory and hospital-based settings. A small number of studies reported findings in cohorts of adults (*n* = 7). The number of studies of pediatric hospital cohorts and pediatric ambulatory cohorts were similar (*n* = 16 and *n* = 20, respectively). Among pediatric hospital cohorts, most studies (81%, 13/16) were conducted exclusively in intensive care units and three studies included pediatric patients from intensive care units and non-intensive care unit hospital wards (Supplementary Table 1). Twelve pediatric hospital studies were restricted to infants alone.

**Fig. 3:**
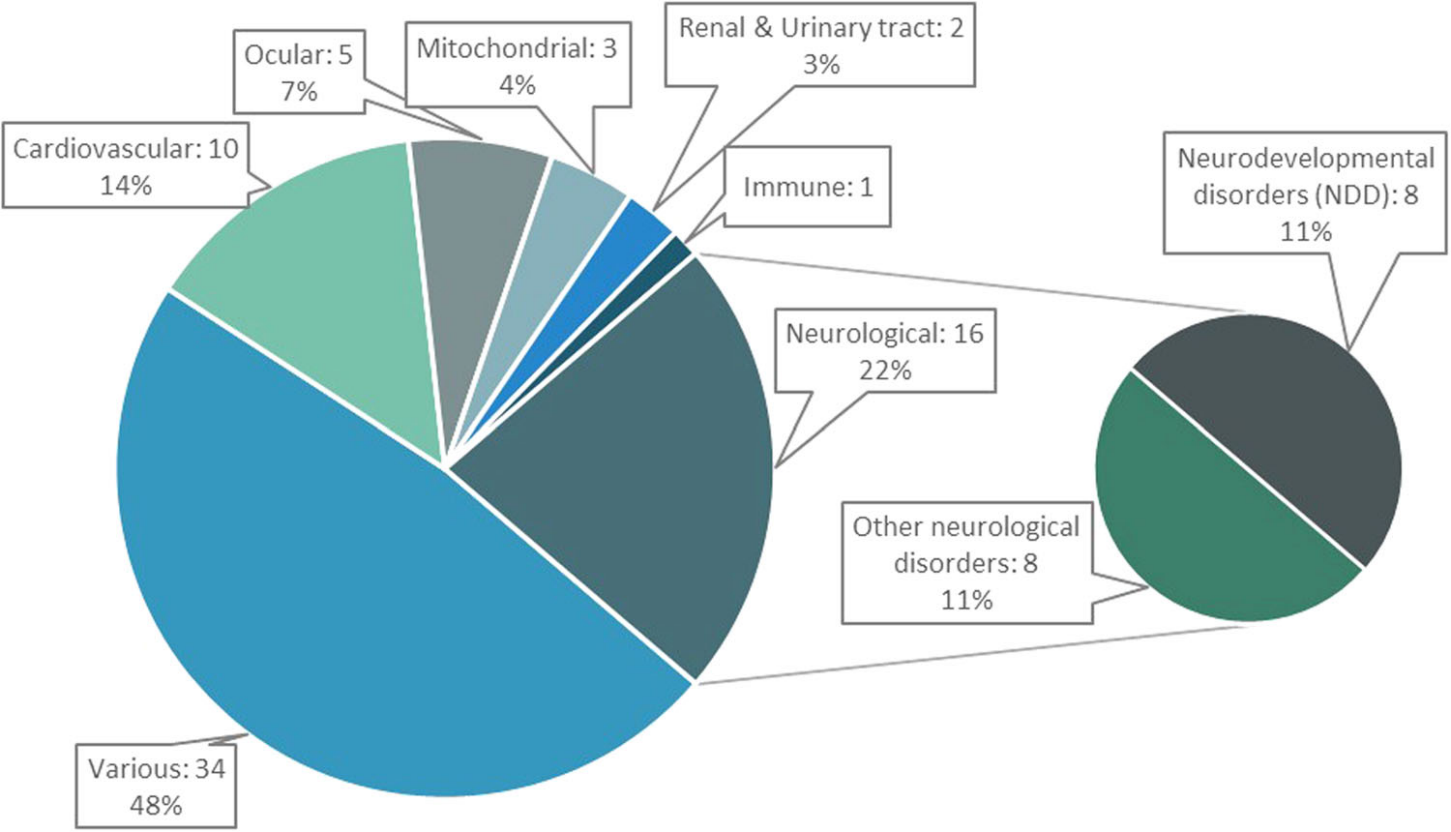
Phenotype categories. Summary of indications for testing of included GS studies (total of 71 studies). Studies involving primary neurological disorders were further classified into those involving neurodevelopmental disorders (NDD) or those involving other neurological phenotypes. All cohorts in the NDD category involved individuals with developmental delay (DD) or intellectual disability (ID) and included studies where cohorts had conditions such as autism spectrum disorder or epilepsy in addition to DD/ID. Heterogeneous cohorts refer to studies where individuals had symptoms or signs concerning a genetic disorder that were not limited to a specific phenotype or single organ system.

Nearly half of the studies were performed in phenotypically heterogeneous study cohorts, where participants either had signs or symptoms suggestive of a rare genetic disorder and were not limited to a specific phenotype (e.g., epileptic encephalopathy) or single organ system (*n* = 34, see Fig. 3). This was followed by study cohorts composed exclusively of neurological (*n* = 16), cardiovascular (*n* = 10), ocular (*n* = 5), and renal and urinary tract (*n* = 2) disorders. Most pediatric hospital studies involved heterogeneous phenotypes (*n* = 14/16), with the remaining studies focused on patients with cardiovascular or neurological disorders. Of note, all seven of the studies involving adult ambulatory patients focused on organ-specific phenotypes (cardiovascular *n* = 4, ocular disorders *n* = 1, renal and genitourinary disease *n* = 1, neurological *n* = 1, immune *n* = 1).

### Diagnostic yield

**Fig. 4:**
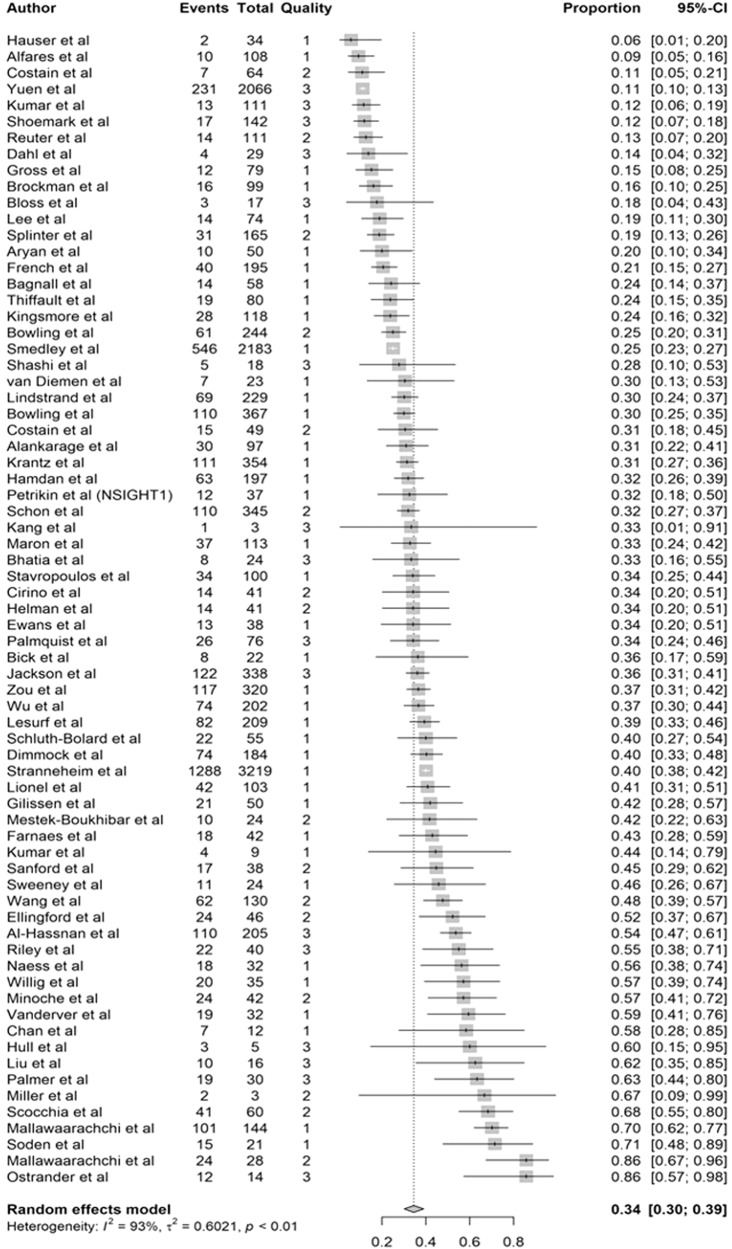
Comparison of diagnostic yield across studies.

Across all indications and care settings, diagnostic yield ranged from 6–86% (See Supplementary Fig. 1). The unweighted mean diagnostic yield was higher when GS was applied as a first-line genetic test (45%, range 12–73%) compared to cohorts who had previously received genetic testing (33%, range 6–86%) or in those with negative prior ES (33%, range 9–60%, see Table 3). From the random effects model of proportions, we generated a point estimate for the mean diagnostic yield across the 71 studies that accounts for cohort size and the number of diagnosed cases per cohort. This point estimate for diagnostic yield was 0.34 (95% CI 0.3012-0.3900). Diagnostic yield varied considerably across studies (tau^2^ 0.6021, I^2^ 93%, 95% CI 92 to 94%, see Fig. 4).

**Table 3:** Unweighted diagnostic yield and clinical utility based upon extent of prior genetic testing

	Number of studies	Mean diagnostic yield % (range)	Number of studies reporting clinical utility	Mean clinical utility % (range)
First-line GS	27	45 (12–73)	16	49 (13.1–100)
Prior genetic tests (ES in <80%)	36	33 (6–86)	6	32 (20.4–65)
ES-negative (ES in >80%)	8	33 (9–60)	1	75* (N/A)
Total	71	33.6 (6–86)	23	

*GS* short read whole genome sequencing, *ES* whole exome sequencing.

*Represents only one study.

### Clinical utility or benefit

Overall, 32% of the studies (23/71) reported on quantitative measures of clinical utility or benefit. Measures of clinical utility assessed one or more of the dimensions of utility as described by Hayeems and colleagues^25^: diagnostic thinking efficacy, therapeutic efficacy, patient outcome efficacy, and society efficacy. Among these studies, 70% (16/23) applied GS as a first-line test. When comparing by care setting, the clinical utility was most frequently reported in studies of hospitalized pediatric patients (14/16). Twenty-three studies reported changes in management as a result of a molecular etiology identified by GS. The proportion of patients who experienced a change in management varied among study cohorts (20–100%). Changes in management included both acute changes to treatment (e.g., medication or dietary change, other diagnostic laboratory and imaging testing) as well as long-term changes (e.g., disorder-associated surveillance). In addition to changes in management based on a new molecular diagnosis, two studies^26,27^ described changes in management for patients with non-diagnostic GS (7–16%). Other dimensions of clinical utility, such as patient outcome efficacy (e.g., do patients who receive GS have better outcomes than those who do not) or societal efficacy (e.g., cost-effectiveness, testing acceptable to society), were described less frequently (8/23, 9/23, respectively).

### First-line GS

GS was deployed as the first diagnostic genetic test in 38% (27/71) of studies. First-line GS testing was most commonly implemented in pediatric hospital-based settings (48% or 13/27 studies), whereas adult and pediatric ambulatory and mixed cohorts were more frequently reported to have undergone some genetic testing prior to GS. Most studies (56%,15/27) of first-line GS involved patients with heterogeneous phenotypes. Phenotype-specific studies of first-line GS included cardiovascular disorders (*n* = 3), neurological and/or neurodevelopmental disorders (*n* = 4), ocular disorders (*n* = 3), and renal and/or genitourinary tract disorders (*n* = 2). Clinical utility was reported in 16 studies (59%) and most of these studies (13/16) involved pediatric hospital-based settings. Changes in management associated with molecular diagnosis identified by GS were reported in all studies and varied in frequency by cohort selection and underlying molecular diagnosis (24–100%).

### Study quality

Assessment of study quality by American College of Radiology (ACR) criteria^28^ determined that 39 studies were classified as category 1 (highest quality), 16 studies as category 2 (medium quality), and 16 studies as category 3 (modest quality). Study designs varied and included registered clinical trials, single or multi-center prospective cohort studies, and retrospective cohort studies. With the exception of the registered clinical trials, most studies provided abbreviated descriptions of inclusion and exclusion criteria for study inclusion.

### Ratings rounds

The findings of this literature review were summarized and provided to the expert panel and informed the development of patient selection recommendations.

Several ratings rounds were performed with the level of consensus increasing with each round (Supplemental Results). Five core “points to consider” were developed.

### Points to consider

The following recommendations were developed by a working group of the Medical Genome Initiative based on evidence from the focused literature review and expert opinion. These recommendations are intended to provide a framework for selecting patients, both pediatric and adult, with a suspected genetic disorder for first-line clinical GS testing. These recommendations are not intended to replace individual clinician judgment. As the cost of GS technology continues to decline, and additional gene-disease relationships are described, GS testing will be appropriate for a wide range of clinical indications which are not addressed here.

1. **We recommend GS as the first-line genetic test for pediatric patients in intensive care units with an unexplained illness with a possible genetic etiology. Rapid GS should be considered in this setting**. While there is no accepted standard definition for rapid GS, providers should consider weighing the expected time to test results with the need for time-sensitive clinical decision-making.
2. **We recommend GS when sequential genetic tests are being considered because the patient’s features indicate a likely genetic cause, but do not suggest a single recognizable disorder**. Examples include multiple congenital anomalies or syndromic intellectual disability when a specific disorder is not clinically identified.
3. **We recommend GS when current panel testing does not encompass all the variants that are known to be causative of a disorder**. Examples of variant types include coding and non-coding SNVs, small indels, CNVs, short tandem repeat expansions, copy-neutral structural variants, and mt-DNA variants. As a result, the clinician is considering sequential targeted genetic tests to comprehensively evaluate relevant variant types for the disorder in question. For example, current gene panels for intellectual disability may include coding SNVs, small indels but may not be specifically validated for the detection of certain structural variants or provide sufficient resolution to detect large CNV breakpoints.
4. **We recommend GS when patients are being treated for a non-genetic condition but have a clinical course and/or response to therapy that may be better explained by a rare genetic diagnosis**. Examples may include an unusually severe or prolonged clinical course, atypical response, or failure to respond to standard therapy. In addition, the clinician may wish to consider other types of genetic variation, such as pharmacogenomic alleles, that may also contribute to response to therapy and can be detected by GS.
5. **We support targeted genetic testing as an alternative to first-line GS when an individual’s features strongly suggest a single recognizable genetic disorder and the clinician determines that targeted genetic testing is likely to identify the disorder**. Based on the individual clinician’s judgment, the potential for phenotypically similar disorders, and the specificity of the patient’s features for the disorder in question, the clinician may still opt for a more comprehensive first-line test such as GS. Examples of targeted genetic testing include single or limited panel of genes for a specific disorder, such as *FGFR3* genotyping for achondroplasia, karyotype for suspected Trisomy 21, repeat expansion testing of *HTT* for Huntington’s disease, panels for non-syndromic retinitis pigmentosa or hypertrophic cardiomyopathy. Current analytic capabilities of GS can detect these variant types, however, due to current resource constraints, targeted genetic testing may be a more cost-effective alternative strategy. As sequencing costs continue to decline, the relative cost-effectiveness of first-line GS for such scenarios may improve over time. Targeted testing could also be considered where features suggest a specific disorder (e.g., Lynch syndrome) and one or more of the associated genes (e.g., *PMS2)* occur in a region with high sequence homology that may be challenging to detect with short read GS or a greater depth of coverage is required to detect a suspected mosaic disorder. Clinicians may wish to consider the most appropriate tissue type to sequence when the specific disorder in question is known to have variable mutation load in the blood and an alternative tissue type may be preferred for detection (e.g., fibroblasts for a possible mosaic disorder or muscle for certain mitochondrial disorders associated with higher levels of heteroplasmy in muscle).

## Discussion

Early use of GS can shorten the diagnostic odyssey experienced by many patients with rare genetic disorders, which is essential as more targeted therapies emerge. GS has robust diagnostic capabilities to identify rare genetic disorders through simultaneous analysis of numerous genetic loci and variant types. The diversity of phenotypes and care settings where GS has been studied to date highlight the many potential applications of GS to various healthcare settings and patient populations. These studies demonstrate that first-line GS reduces the time to diagnosis and treatment for rare genetic disorders, thereby streamlining healthcare utilization and costs, and, importantly, reducing suffering to affected individuals and their families. Furthermore, first-line GS has the potential to reduce healthcare inequities by enabling diagnostic equity^29^.

In this analysis, we aimed to appraise the current evidence for use of first-line GS across a variety of clinical settings and indications. Many healthcare systems and payers face resource constraints that limit more widespread use of first-line GS. Understanding the specific clinical scenarios where first-line GS may yield the most benefit can therefore inform the initial indications where use would be most advantageous. We first aggregated the 71 studies into four clinical settings that share similar patient populations, clinical urgency, acuity, and likely similar healthcare resource constraints (pediatric hospital cohorts, pediatric ambulatory care cohorts, mixed cohorts, and adult cohorts).

To date, the majority of studies have evaluated hospitalized pediatric patients or have been mixed settings (combining hospitalized and ambulatory settings). These groups include multiple high-quality studies and the majority of the investigations in this setting applied GS as a first-line genetic test. Moreover, these studies reported frequent changes in management for those patients who received positive clinical GS test findings, demonstrating that GS testing supports treatment planning decisions. The data from multiple large clinical trials of genomic sequencing and observational cohort studies robustly support that first-line GS is effective to diagnose rare genetic disorders in high-acuity pediatric populations, such as pediatric patients that are hospitalized or in intensive care units. Rapid testing is appropriate in such populations where medical urgency necessitates timely results to facilitate diagnosis and treatment planning.

In other clinical scenarios, GS shows promise but there is limited evidence to recommend the first-line use to diagnose rare genetic disorders across diverse phenotypic presentations and care settings without the involvement of genetic experts. Moreover, there are limitations to the published literature that are important to consider. First, limited numbers of high-quality studies and small sample sizes preclude more granular analyses of certain clinical settings and phenotype-specific indications (e.g., mitochondrial disorders, congenital heart disease). Second, there are substantial methodological differences among studies. These differences include variations in study design, inclusion and exclusion criteria, outcomes measures reported and employed, and GS analysis (including family sequencing strategy, analytical pipeline, reporting criteria). Such methodological differences may have varying impacts, which include (1) diagnostic yield may be affected by variability in the analytical pipeline employed; (2) differences in clinical utility outcome measures make meta-analyses across studies intractable; and (3) potential for uncorrected ascertainment biases inherent to some study designs and inconsistency in inclusion and exclusion criteria. Thus, while first-line GS shows promise in other clinical settings beyond hospitalized infants and children in intensive care units, additional studies are recommended to further inform evidence-based recommendations in other settings.

This review informs the future actions that are imperative to address the current evidence gaps and refine indications for the use of first-line GS. Additional high-quality studies are essential to further delineate the utility of first-line GS in comparison to current clinical practice, such as referral for specialist clinical evaluation with or without conventional genetic testing for phenotype-specific indications or specific clinical populations (e.g., adult populations). Attention to population selection, recruitment, and consistent application of inclusion and exclusion criteria are also essential to limit potential ascertainment biases. Studies of GS in healthcare settings should include the comparison to standard of care (which may include no genetic testing, which may be the case for some adult-onset conditions such as certain cardiovascular diseases) and consistent measures of clinical utility (for further discussion, consider the study by ref. 25). Additional longitudinal studies are needed to further assess if near-term changes in management result in improved and durable long-term outcomes and cost-savings.

Assessment of the clinical utility of first-line GS is essential in future studies. In this focused literature review, only 32% of studies evaluated the clinical utility of sequencing. Among those that did, there was considerable variability in how clinical utility was measured and reported. Most of these studies primarily reported on changes in management for the individual tested with fewer studies addressing other aspects of clinical utility, including impacts for other family members or cost-effectiveness. Furthermore, reports of utility focused on acute changes in management may not reflect long-term benefits such as avoiding additional diagnostic testing (e.g., a sedated diagnostic imaging test in a pediatric patient). While individual case reports of diagnostic GS did not meet the inclusion criteria for this literature review, these reports highlight the utility of GS described within the larger cohort study publications. A summary of 32 case reports of diagnostic GS identified during this literature review are listed in Supplementary Table 5, including multiple cases where acute care interventions are likely to have prevented premature death or long-term morbidity.

It should be noted that perceived clinical utility for immediate care may be influenced by the time to test results. There is currently no standard for turn-around time for clinical or even “rapid” GS testing, though it is suggested that faster turn-around times are more likely to lead to therapeutic interventions with sustained clinical benefit and cost-savings^27^.

Diagnostic yield was the most consistently reported outcome among studies included in this review. The unweighted diagnostic yield of GS varied substantially across 71 studies (Supplementary Fig. 1) and was observed to be qualitatively higher with first-line GS (45%) and similar in cohorts with prior genetic testing (33%) and exome-negative cohorts (33%). Studies varied in the level of detail provided on the factors which may contribute to diagnostic GS findings in cohorts with prior genetic testing or ES and include additional variant types not detected by prior testing, established disease genes not included on targeted tests, multiple distinct rare genetic diseases identified within the same individual after detection of additional pathogenic variants not identified on first tier testing, novel disease genes, or increased/additional evidence for a variant previously detected but not interpreted as diagnostic. Additional studies comparing other diagnostic interventions with GS within individuals may further inform the relative variant and technical contributions to the diagnostic efficacy of GS. For example, recently published recommendations for best practices in the sequencing and interpretation of GS may improve consistency among laboratory practices, particularly among certified clinical laboratories^15,30^. Ongoing developments specific to other difficult-to-detect or interpret variant types, including copy-neutral structural variants, variants in regions of high sequence homology, non-coding variation^31–33^ will continue to improve the diagnostic efficacy of GS. Incorporation of these advancements into routine clinical GS, combined with consistent evaluation of the utility of GS for specific phenotypes is essential to further inform the efficacy of GS for rare genetic disorders in comparison to other conventional genetic tests or untargeted methods. Moreover, while diagnostic yield informs the capabilities of GS as a diagnostic test, it does not inform the utility of GS in healthcare, including changes in management for individual patients, identification of other at-risk family members, or cost-effectiveness.

Our findings align with the results of recent systematic reviews and meta-analyses of the utility of ES and GS^14,34,35^ to diagnose rare genetic disorders. Furthermore, a recent systematic review and guideline by the American College of Medical Genetics and Genomics (ACMG)^12^ supports the use of GS for individuals with selected phenotypes, including those with congenital anomalies and intellectual disability. These studies differed in study/cohort inclusion criteria and by combining the findings of ES and GS studies into analyses^14,35^ thereby lending more general evidence to untargeted diagnostic sequencing. In contrast, the meta-analysis by Chung and colleagues^21^, which compared the diagnostic rate and utility of ES and GS, as well as our findings provide additional support for GS as an untargeted diagnostic test.

Many healthcare systems face resource constraints that preclude more widespread use of GS. These include cost and reimbursement constraints as well as a limited number of available clinicians to support the coordination, counseling, and interpretation of GS. Identifying patients for first-line GS may enable testing for patients most likely to benefit. Access to virtual reference systems and other educational resources as well as availability of remote consultation and counseling services, will likely be a key to equip front-line clinicians in the practice of genome-informed medicine^36,37^. The available evidence currently supports the use of GS as a first-line test for hospitalized pediatric patients in intensive care units where the efficiency of the diagnostic assessment for rare genetic disorders facilitates real-time clinical decision-making in patients with life-threatening illness. Further study comparing test performance, clinical utility, and cost-effectiveness with other first-line genetic tests (e.g., chromosomal microarray, NGS panels) and other untargeted diagnostic sequencing approaches such as exome sequencing in non-acute care settings is recommended. This would further delineate the appropriateness of GS as a first-line test under current resource constraints where testing remains important but may not be medically urgent. There are more than 700 childhood-onset treatable genetic disorders, therefore the need for rapid diagnosis, even in the outpatient setting should not be underestimated (https://www.rx-genes.com/about/). Harnessing study design and inclusion criteria that emulate real-world clinical practice, particularly for phenotype-specific indications, are key to further delineate the evidence in these scenarios. As sequencing costs continue to decline, the use of GS as a first-line genetic test rather than a test of last resort becomes more feasible. Early use of GS can facilitate timely diagnosis which is crucial as more targeted therapies for rare genetic disorders emerge.

## Methods

An expert panel of Medical Genome Initiative members was formed in 2020 to develop evidence-based recommendations regarding patient prioritization for first-line GS. Most panel members were healthcare providers (i.e., board certified medical geneticists, genetic counselors, genetics nurses) but also included clinical genetics laboratory directors from CLIA/CAP certified laboratories. All panel members had significant experience in diagnostic genetic testing, including GS.

The panel elected to use the American College of Radiology (ACR) Appropriateness Criteria (AC)^28^ methodology, given the notable similarities between diagnostic imaging (e.g., MRI) and genetic testing. The process was also guided by the recently published American College of Genetics and Genomics systematic evidence-based review^12^, which sought to determine the clinical utility of ES/GS in pediatric patients with congenital anomalies or intellectual disability. Recommendation development was driven by two elements: (1) a systematic analysis of the peer-reviewed literature and (2) an assessment of the appropriateness of GS in a broad range of clinical indications guided by panel expertize.

### Systematic literature search and analysis

To answer the overarching question of “In which patient populations would first-tier GS be the most appropriate and beneficial for obtaining a genetic diagnosis?”, the panel used the PICOS (population, intervention, comparator, setting) framework to guide their search strategy.

#### Population

Studies with patients undergoing GS for diagnosis of genetic disease were included. Similar to ref. 14, our search included a broad range of genetic rare disease indications and was not limited by study cohort age. Application of GS in the following scenarios were excluded: prenatal/fetal, healthy genomes (screening), oncology, and microbiology/infectious disease.

#### Intervention

GS

#### Comparator

Usual care (which included both studies where patients received another genetic test such as chromosomal microarray or ES as well as studies where usual care included no other genetic testing.

#### Outcomes

Studies had to include an assessment of at least one of the following outcomes for inclusion: diagnostic yield, clinical utility/benefit, or both. Diagnostic yield was defined as the number of patients in whom a molecular genetic diagnosis was achieved with GS divided by the total number of patients who received GS. We adopted our definition of clinical utility or benefit from a previous Medical Genome Initiative publication^4^ which included measures of diagnostic thinking efficacy (i.e., Did GS strengthen or weaken a clinicians hypothesis about molecular etiology?), therapeutic efficacy (i.e., Did GS alter the patient’s clinical care pathway or medical management?), patient outcome efficacy (i.e., Did GS improve patient health, offer personal utility or improve psychosocial well-being?) and societal efficacy (i.e., Is GS cost-effective? Were there family or whole population impacts?).

#### Setting

Studies conducted in a clinical or research setting.

### Search strategy

The search strategy was developed by a literature review core team and approved by the panel. Two electronic databases (i.e., PubMed and Dimensions) were queried to ensure the most comprehensive search results. The searches were initially performed on 12 March 2020 and updated on 01 August 2022. Studies published between 1 January 2011 to 01 August 2022 were included. The searches were limited to studies written in English with human subjects using the following search string: (genome sequencing) or (whole genome sequencing) and (“diagnosis” or “clinical”) and “genetic disease” NOT (genome-wide) NOT (cancer) NOT (oncology) NOT (prenatal).

References were imported into DistillerSR, a web-based systematic literature review management program. The duplicate detection feature allowed us to remove articles found by both electronic databases. Title and abstract screening were performed by a single reviewer to exclude articles not meeting inclusion criteria. The following exclusion criteria were applied: lack of assessment of diagnostic yield or clinical utility, inappropriate publication type (e.g., reviews, editorials, conference abstracts), and inappropriate study design (e.g., animal studies, WES-only studies, case reports). Case reports and case series represent valuable and important pieces of evidence, and although they were technically excluded from the review, a separate repository was created to keep track of them. These can be found in Supplementary Table 5.

### Data extraction

Each reference that moved on to the full-text review and data extraction phases was reviewed by two independent reviewers. Custom data extraction forms were created in DistillerSR to collect a broad range of information related to study characteristics (e.g., study setting and patient cohort) and outcome data (e.g., diagnostic yield). A detailed list of information gathered during initial data extraction can be found in the Supplementary Methods—Data Extraction Form. Further data extraction was subsequently conducted to obtain additional details on GS analysis. Discordance between reviewers was resolved through regular meetings and discussions.

### Assessing study quality

A determination of study quality was performed using the ACR AC method^28^. Briefly, studies were assigned to a category (1–3) based on the study type and an assessment of the predefined and equally weighted quality components (See Supplementary Methods—Study Quality Components). The size of the study was not included in the determination of the study quality. Category 1 and 2 studies were regarded as well-designed studies that accounted for common biases, whereas Category 3 studies were likely to have study design limitations. High-quality studies were defined as category 1 or 2.

### Data analysis

The data were exported from DistillerSR to Microsoft Excel to generate descriptive statistics and summary tables and figures. The overall average unweighted diagnostic yield was calculated by adding the yield of all the studies and dividing by the total number of studies. A bubble plot was created to visualize the diagnostic yield range across phenotypic groups inclusive of study size. A pie chart was constructed to assess the variety of clinical phenotypes represented across all studies. Summary tables grouped by cohort (i.e., Adult Cohorts, Pediatric Hospital Cohorts, Pediatric Ambulatory Cohorts, and Mixed Cohorts).

To obtain an estimate of the overall diagnostic rate among all the studies and within specified subgroups, we used a random effects model of proportions. A generalized linear mixed model was estimated after logit transformation of the proportion of positive tests to total probands in each study^38^. Confidence intervals were estimated using the Clopper–Pearson exact binomial method. We used the year of publication as a numerical variable in a meta-regression model. Subgroup analyses were performed for several categorical variables, including phenotype group, quality score, study population, study design, cohort test characteristics, and testing strategy. All calculations were performed in R using the meta package^39^.

### Evidence summaries and recommendations

A sub-team was formed to develop a preliminary set of recommendations based on the data gathered from the systematic evidence review. Evidence tables for each recommendation were created, which contained a summary of the supporting references identified through the systematic review.

### Appropriateness rating rounds for recommendations

To assess how strongly the panel supported the recommendations and to achieve consensus, a series of ratings rounds were performed based on a modified ACR AC^28^ appropriate use methodology which is based on the Delphi method. First, members were asked to rate their level of agreement with each recommendation using the evidence tables described above and their personal clinical experience. This information was gathered using a web-based questionnaire where panel members were asked to rank their support on a nine-point Likert scale (1 – strongly disagree, 9 – strongly agree). Consensus was achieved when all panel members ranked each recommendation as either 8 (agree) or 9 (strongly agree). The questionnaire also contained free text comment boxes to capture individual feedback. Consistent with ACR AC algorithms, raters were asked to assume the following:

- The primary assessment for diagnostic testing is the diagnostic utility, diagnostic accuracy, test performance, etc., of performing GS as a first-tier test
- The patients do not have contraindications for GS
- GS is available and accessible to all
- GS is performed and results are interpreted by experts
- The direct or indirect costs of GS are not considered as a benefit or harm when determining appropriateness.

When a recommendation failed to reach the level of consensus, the panel members were gathered to discuss the anonymized results and to resolve discrepancies. The panel was then asked to rank their level of agreement with the revised recommendation. This process of revising and ranking was repeated until a consensus was reached. The results of the ratings rounds for each recommendation can be seen in the Supplemental Results.

### Reporting summary

Further information on research design is available in the Nature Research Reporting Summary linked to this article.

### Supplementary information

Supplementary File

REPORTING SUMMARY

## Data Availability

The datasets generated and/or analyzed during the current study are available in the GitHub repository, https://github.com/MedicalGenomeInitiative/Patient-Selection.

## Code availability

Code and raw data files are available at https://github.com/MedicalGenomeInitiative/Patient-Selection. The following software versions were used: RStudio 2023.09.0 Build 463, R version 4.1.2 (2021-11-01)—“Bird Hippie,” Quarto version 1.3.433. Variales and statistical function non-default parameters are specified in the *.qmd file.
