# Supplementary material for: Evidence review and considerations for use of first line genome sequencing to diagnose rare genetic disorders
Source: NPJ Genom Med. 2024 Feb 26;9:15. doi: 10.1038/s41525-024-00396-x (PMC10897481; doi:10.1038/s41525-024-00396-x)
Supplement: Supplementary file 1 — Supplementary File [file 41525_2024_396_MOESM1_ESM.pdf]

Supplementary Information

Supplementary Table 1

Supplementary Table 2

Supplementary Table 3

Supplementary Table 4

Supplementary Table 5

Supplementary Figure 1

Supplementary Methods

Supplementary Results

Supplementary References

Supplementary Table 1. Studies in Pediatric Hospital Patient Cohorts

Reference (PMID)	Year	Study Design	Country	Probands (N)	Clinical Phenotype	Level of Prior Genetic Testing	Family testing strategy	Initial analysis strategy	DY (%)	COM (%)	Study Quality Category
Krantz et al[1] (34570182)	2021	Clinical Trial*	United States	354	Heterogeneous	First-line test	Mixed	Untargeted	31.3	24.2	1
Maron et al[2] (33587123)	2021	Clinical Trial*	United States	113	Heterogeneous	First-line test	Mixed	Untargeted	33	78	1
NSIGHT2 (Kingsmore et al, 2019[3], Dimmock et al 2020[4] & Cakici et al 2020[5]) (31564432)	2019 & 2020	Clinical Trial	United States	118 (GS), 95 rWES (for 213 overall)	Heterogeneous	First-line test	Proband-only	Untargeted	24	32.5 (63% in UR-GS)	1
NSIGHT1 Trial (Petrikin et al 2018[6] & Berrios et al 2019[7])	2018	Clinical Trial	United States	37	Heterogeneous	First-line test	Mixed	Untargeted	32	48	1
Bowling et al[8] (34930662)	2022	Prospective cohort*	United States	367	Heterogeneous	Some tests (ES in <80%)	Trio	Untargeted	30		1
Dimmock et al[9] (34089648)	2021	Prospective cohort*	United States	184	Heterogeneous	First-line test	Mixed	Untargeted	40	32	1
Sweeney et al[10] (33888711)	2021	Prospective Cohort	United States	24	Cardiovascular-CHD	First-line test	Mixed	Untargeted	46	100	1
Wang et al[11] (31965297)	2020	Prospective Cohort	China	130	Heterogeneous	First-line test	Trio	Untargeted	47.7	48.3	2
Sanford et al[12] (31246743)	2019	Prospective Cohort	United States	38	Heterogeneous	Some tests (ES in <80%)	Mixed	Untargeted	45	24	2
Hauser et al[13] (29368431)	2018	Prospective cohort	United States	34	Cardiovascular-CHD	Some tests (ES in <80%)	Trio	Panel	6		1
Mestek-Boukhibar et al[14] (30049826)	2018	Prospective cohort	UK	24	Heterogeneous	First-line test	Trio	Untargeted	42	33	2
van Diemen et al[15] (28939701)	2017	Prospective cohort	Nether-lands	23	Heterogeneous	First-line test	Trio	Untargeted	30	71.4	1
Wu et al[16] (33935161)	2021	Prospective cohort*	China	202	Heterogeneous	First-line test	Trio	Untargeted	36.6	21.6	1
Palmquist et al[17] (35115709)	2022	Retrospective Cohort	United States	76	Heterogeneous	First-line test	Trio	Untargeted	34	77	3
Farnaes et al[18] (29644095)	2018	Retrospective cohort	United States	42	Heterogeneous	First-line test	Trio	Untargeted	43	31	1
Willig et al[19] (25937001)	2015	Retrospective cohort	United States	35	Heterogeneous	First-line test	Trio	Untargeted	57	65	1

CHD: congenital heart disease; COM: Change in Management; DY: diagnostic yield; ES: whole exome sequencing; GS: whole genome sequencing; PMID: Pubmed Identification number; rWES: rapid whole exome sequencing; UK: United Kingdom; UR-GS: ultra-rapid whole genome sequencing

*Multi-center study

Supplementary Table 2. Studies in Pediatric Ambulatory Care Cohorts

Reference (PMID)	Year	Study Design	Country	Probands (N)	Clinical Phenotype	Level of Prior Genetic Testing	Family testing strategy	Initial analysis strategy	DY (%)	COM (%)	Study Quality Category
Palmer et al[20] (33568551)	2021	Prospective cohort	Australia	30	Neuro-NDD	Some tests (ES in <80%)	Trio	Untargeted	53		3
Zou et al[21] (34145886)	2021	Prospective cohort	China	320	Neuro-Other	First-line test	Proband-only	Untargeted	36.6	13.1	1
Al-Hassnan et al[22] (32870709)	2020	Prospective Cohort	Saudi Arabia	205	Cardiovascular-Cardiomyopathy	First-line test	Proband-only	Untargeted	53.7		3
Costain et al[23] (32960281)	2020	Prospective cohort	Canada	49	Heterogeneous	Some tests (ES <80%)	Trio	Untargeted	30.6	20.4	2
Jackson et al[24] (32830442)	2020	Prospective cohort*	UK	338^	Ocular	First-line test	Mixed	Panel	35.6		3
Reuter et al[25] (32037394)	2020	Prospective Cohort	Canada	111	Cardiovascular-CHD	Some tests (ES in <80%)	Trio	Untargeted	12.6		2
Riley et a[26] (32313153)	2020	Prospective cohort*	Australia	40	Mitochondrial	Some tests (ES in <80%)	Trio	Untargeted	55		3
Scocchia et al[27] (30792901)	2019	Prospective cohort	United States	60	Heterogenous	First-line test	Trio	Untargeted	68	48	2
Hull et al[28] (31077665)	2019	Prospective Cohort	UK	5	Ocular	Exome-negative (ES >80%)	Mixed	Panel	60		3
Thiffault et al[29] (30008475)	2019	Prospective cohort	United States	80	Heterogeneous	Some tests (ES in <80%)	Mixed	Untargeted	24		1
Alankarage et al[30] (30293987)	2019	Prospective cohort*	Australia	97	Cardiovascular-CHD	Some tests (ES in <80%)	Mixed	Panel	31		1
Ostrander et al[31] (30109124)	2018	Prospective Cohort	United States	14	Neuro-Other	Some tests (ES in <80%)	Trio	Panel	86		3
Costain et al[32] (29453418)	2018	Prospective cohort	Canada	64	Heterogeneous	Some tests (ES in <80%)	Proband-only	Untargeted	11		2
Lionel et al[33] (28771251)	2018	Prospective cohort	Canada	103	Heterogeneous	Some tests (ES in <80%)	Proband-only	Untargeted	41		1
Bowling et al[34] (28554332)	2017	Prospective cohort	United States	244	Neuro-NDD	Some tests (ES in <80%)	Mixed	Untargeted	25		2
Yuen et al[35] (28263302)	2017	Prospective cohort*	Canada	2066 unique families	Neuro-NDD	Some tests (ES in <80%)	Trio	Untargeted	11.2		3
Bick et al[36] (28496993)	2017	Prospective cohort	United States	22	Heterogeneous	Exome-negative (ES >80%)	Mixed	Untargeted	36	75	1
Stavropoulos et al[37] (28567303)	2016	Prospective cohort	Canada	100	Heterogeneous	First-line test	Proband-only	Untargeted	34		1

Reference (PMID)	Year	Study Design	Country	Probands (N)	Clinical Phenotype	Level of Prior Genetic Testing	Family testing strategy	Initial analysis strategy	DY (%)	COM (%)	Study Quality Category
Lesurf et al[38] (35288587)	2022	Retrospective cohort	Canada	209	Cardiovascular-cardiomyopathy	Some tests (ES in <80%)	Mixed	Panel	39		1
Miller et al[39] (27884935)	2017	Retrospective cohort	UK	3	Other-craniosynostosis	Some tests (ES in <80%)	Trio	Panel	66		2

CHD: congenital heart disease; COM: Change in Management; DY: diagnostic yield; ES: whole exome sequencing; NDD: Neurodevelopmental disorders; PMID: Pubmed Identification number; UK: United Kingdom;

*Multi-center study

^Jackson et al, 2 cohorts included: single clinical center consecutive patient series (45 probands who received WGS) and cohort from UK 100,000 genomes project (293 probands)

Supplementary Table 3. Studies in Mixed Patient Care Settings

Reference (PMID)	Year	Study Design	Country	Probands (N)	Clinical Phenotype	Level of Prior Genetic Testing	Family testing strategy	Initial analysis strategy	DY (%)	COM (%)	Study Quality Category
Brockman et al[40] (33976420)	2021	Clinical Trial	United States	99	Heterogeneous	First-line test	Proband-only	Untargeted	16		1
Vanderver et al[41] (32342562)	2020	Clinical Trial*	United States	32	Neuro-Other	First-line test	Trio	Untargeted	59.3		1
Ewans et al[42] (35970915)	2022	Prospective Cohort	Australia	38	Heterogeneous	Exome-negative (ES >80%)	Trio	Untargeted	34		1
Bhatia et al[43] (32819910)	2021	Prospective cohort*	Singapore	24	Heterogeneous	Some tests (ES in <80%)	Trio	Untargeted	33.3	27	3
Chan et al[44] (33808351)	2021	Prospective Cohort	Australia	12	Ocular	First-line test	Proband-only	Panel	59.3		1
Stranneheim et al[45] (33726816)	2021	Prospective Cohort	Sweden	3219	Heterogeneous	First-line test	Proband-only	Panel	40		1
Mallawaarachchi et al[46] (33437033)	2021	Prospective Cohort	Australia	186	Other-Renal and Urinary Tract	First-line test	Proband-only	Panel	47.9		1
Schon et al[47] (34732400)	2021	Prospective cohort*	UK	319	Mitochondrial	Some tests (ES in <80%)	Mixed	Panel	31		2
Dahl et al[48] (32040484)	2020	Prospective cohort	Sweden	29	Ocular	First-line test	Proband-only	Untargeted	14		3
French et al[49] (30847515)	2019	Prospective cohort	UK	195	Heterogeneous	Some tests (ES in <80%)	Mixed	Untargeted	21	65	1
Kumar et al[50] (31731261)	2019	Prospective cohort*	Australia	111	Neuro-Other	First-line test	Mixed	Panel	11.7		3
Liu et al[51] (31852928)	2019	Prospective cohort*	China	16	Heterogeneous	Some tests (ES in <80%)	Mixed	Untargeted	62.5		3
Lee et al[52] (31607746)	2019	Prospective cohort	US	74	Heterogeneous	Exome-negative (ES >80%)	Trio	Untargeted	18.9		1
Schluth-Bolard et al[53](30923172)	2019	Prospective cohort*	France	55	Neuro-NDD	Some tests (ES in <80%)	Proband-only	Untargeted	40		1
Shashi et al[54] (29907797)	2019	Prospective cohort	United States	18	Heterogeneous	Exome-negative (ES >80%)	Trio	Untargeted	27.7		3
Splinter et al[55] (30304647)	2018	Prospective cohort*	United States	165	Heterogeneous	Some tests (ES in <80%)	Mixed	Untargeted	19	21	2
Bagnall et al[56] (30025578)	2018	Prospective cohort	Australia	58	Cardiovascular-Cardiomyopathy	Some tests (ES in <80%)	Proband-only	Untargeted	24		1
Alfares et al[57] (29565419)	2018	Prospective cohort	Saudi Arabia	108	Heterogeneous	Exome-negative (ES >80%)	Proband-only	Untargeted	9		1
Hamdan et al[58] (29100083)	2017	Prospective cohort*	Canada	197	Neuro-NDD	Some tests (ES in <80%)	Trio	Untargeted	32		1

Reference (PMID)	Year	Study Design	Country	Probands (N)	Clinical Phenotype	Level of Prior Genetic Testing	Family testing strategy	Initial analysis strategy	DY (%)	COM (%)	Study Quality Category
Kumar et al[59] (27679996)	2016	Prospective Cohort	Australia	9	Neuro-Other	First-line test	Mixed	Panel	44		1
Bloss et al[60] (25790160)	2015	Prospective cohort	United States	17	Heterogeneous	Some tests (ES in <80%)	Trio	Untargeted	18		3
Gilissen et al[61] (24896178)	2014	Prospective cohort	Netherlands	50	Neuro-NDD	Exome-negative (ES >80%)	Trio	Untargeted	42		1
Lindstrand et al[62] (36066546)	2022	Retrospective cohort	Sweden	229	Neuro-NDD	Some tests (ES in <80%)	Proband-only	Untargeted	30		1
Naess et al[63] (32827528)	2021	Retrospective cohort	Sweden	32	Mitochondrial	Some tests (ES in <80%)	Mixed	Untargeted	56		1
Smedley et al[64] (34758253)	2021	Retrospective cohort*	UK	2183	Heterogeneous	Some tests (ES in <80%)	Mixed	Untargeted	24.5	25	1
Helman et al[65] (31912665)	2020	Retrospective cohort*	Australia	41	Neuro-Other	Exome-negative (ES >80%)	Trio	Untargeted	34		2
Gross et al[66] (30293986)	2019	Retrospective cohort	United States	79	Heterogeneous	First-line test	Mixed	Untargeted	15		1
Soden et al[67] (25473036)	2014	Retrospective Cohort	United States	21	Neuro-NDD	First-line test	Trio	Untargeted	73^		1

COM: Change in Management; DY: diagnostic yield; ES: whole exome sequencing; NDD: Neurodevelopmental Disorders; PMID: Pubmed Identification number; UK: United Kingdom

*Multi-center study

Studies with Multiple Distinct Cohorts:

[1]French—2 cohorts:1) neonatal and pediatric intensive care units (167 probands) and 2) heterogeneous cohort recruited from neurology and genetics clinics (28 probands, 15% of total cohort)

[2]Bagnall-2 cohorts: 1) prior genetic testing (46 probands) and 2) cohort with first line WGS (12 probands)

[3]Lindstrandt—2 cohorts: 1) prospective first line GS (100 probands) and 2) non-first line test (129 probands) with retrospective analysis compared to cohort receiving chromosomal microarray and Fragile X testing

[4]Soden—included cohort of negative WES (6 probands) and NICU/PICU cohort of rapid WGS (15 probands)

Supplementary Table 4. Adult study cohorts

Reference (PMID)	Year	Study Design	Country	Probands (N)	Clinical Phenotype	Level of Prior Genetic Testing	Family testing strategy	Initial analysis strategy	DY (%)	COM (%)	Study Quality Category
Cirino et al[68] (29030401)	2017	Clinical Trial[+]	United States	41	Cardiovascular-Cardiomyopathy	Some tests (ES in <80%)	Proband-only	Untargeted	31.7		2
Shoemark et al[69] (35728977)	2022	Prospective cohort	UK	142	Other-Immune	Some tests (ES in <80%)	Proband-only	Panel	12		3
Aryan et al[70] (32847406)	2020	Prospective cohort	United States	50	Other-Cardiovascular	Some tests (ES in <80%)	Proband-only	Untargeted	20		1
Minoche et al[71] (29961767)	2019	Prospective cohort	Australia	42	Cardiovascular-Cardiomyopathy	First-line test	Proband-only	Panel	57		2
Ellingford et al[72] (26872967)	2016	Prospective cohort	UK	46	Ocular	Some tests (ES in <80%)	Proband-only	Panel	52		2
Mallawaarachchi et al.[73] (27165007)	2016	Prospective cohort	Australia	28	Other-Renal and Urinary Tract	First-line test	Proband-only	Panel	86		2
Kang et al[74] (30078120)	2019	Retrospective cohort	Australia	3^	Neuro-Other	Some tests (ES in <80%)	Proband-only	Untargeted	33		3

COM: Change in Management; DY: diagnostic yield; ES: whole exome sequencing; NDD: PMID: Pubmed Identification number; UK: United Kingdom

*Multi-center Study

+Participants enrolled in clinical trial (MedSeq); retrospective analysis of sub-cohort of individuals with hypertrophic cardiomyopathy in cardiology cohort who received WGS.

^Cohort study of 80 individuals but only 3 received WGS

Supplemental Table 5. Excluded case report publications

Alvarez-Mora, Maria Isabel; Corominas, Jordi; Gilissen, Christian; Sanchez, Aurora; Madrigal, Irene; Rodriguez-Revenga, Laia . Novel Compound Heterozygous Mutation in TRAPPC9 Gene: The Relevance of Whole Genome Sequencing. Genes. 2021. 12: 557
Qian, Xinye; Wang, Jun; Wang, Meng; Igelman, Austin D.; Jones, Kaylie D.; Li, Yumei; Wang, Keqing; Goetz, Kerry E.; Birch, David G.; Yang, Paul; Pennesi, Mark E.; Chen, Rui . Identification of Deep-Intronic Splice Mutations in a Large Cohort of Patients With Inherited Retinal Diseases. Frontiers in Genetics. 2021. 12: 647400
Schiff, Elena R.; Tailor, Vijay K.; Chan, Hwei Wuen; Theodorou, Maria; Webster, Andrew R.; Moosajee, Mariya . Novel Biallelic Variants and Phenotypic Features in Patients with SLC38A8-Related Foveal Hypoplasia. International Journal of Molecular Sciences. 2021. 22: 1130
Lin, Yung-Feng; Lin, Tzu-Ching; Kirby, Ralph; Weng, Hui-Ying; Liu, Yen-Ming; Niu, Dau-Ming; Tsai, Shih-Feng; Yang, Chia-Feng . Diagnosis of Arboleda-Tham syndrome by whole genome sequencing in an Asian boy with severe developmental delay. Molecular Genetics and Metabolism Reports. 2020. 25: 100686
Savage, Lane T; Adams, Stacie D; James, Kiely N; Chowdhury, Shimul; Rajasekaran, Surender; Prokop, Jeremy W; Bupp, Caleb P . Rapid whole genome sequencing identifies a homozygous novel variant, His540Arg, in HSD17B4 resulting in D-bifunctional protein deficiency disorder diagnosis.. Molecular Case Studies. 2020. 6: mcs.a005496

Bamborschke, Daniel; Özdemir, Özkan; Kreutzer, Mona; Motameny, Susanne; Thiele, Holger; Kribs, Angela; Dötsch, Jörg; Altmüller, Janine; Nürnberg, Peter; Cirak, Sebahattin . Ultra-rapid emergency genomic diagnosis of Donahue syndrome in a preterm infant within 17 hours. American Journal of Medical Genetics Part A. 2020. 185: 90-96
Larrue, Romain; Chamley, Paul; Bardyn, Thomas; Lionet, Arnaud; Gnemmi, Viviane; Cauffiez, Christelle; Glowacki, François; Pottier, Nicolas; Broly, Franck . Diagnostic utility of whole-genome sequencing for nephronophthisis. npj Genomic Medicine. 2020. 5: 38
Sanford, Erica; Wong, Terence; Ellsworth, Katarzyna A.; Ingulli, Elizabeth; Kingsmore, Stephen F. . Clinical utility of ultra-rapid whole-genome sequencing in an infant with atypical presentation of WT1-associated nephrotic syndrome type 4. Molecular Case Studies. 2020. 6: a005470
Stevenson, Mark; Pagnamenta, Alistair T.; Reichart, Silvia; Philpott, Charlotte; Lines, Kate E.; OxClinWGS; Gorvin, Caroline M.; Lhotta, Karl; Taylor, Jenny C.; Thakker, Rajesh V. . Whole genome sequence analysis identifies a PAX2 mutation to establish a correct diagnosis for a syndromic form of hyperuricemia. American Journal of Medical Genetics Part A. 2020. 182: 2521-2528
Kops, Samantha A.; Kylat, Ranjit I.; Bhatia, Shanti; Seckeler, Michael D.; Barber, Brent J.; Bader, Mohammad Y. . Genetic Characterization of a Model Ciliopathy: Bardet–Biedl's Syndrome. Journal of Pediatric Genetics. 2020. :

Wu, Jinyu; Yu, Ping; Jin, Xin; Xu, Xiu; Li, Jinchen; Li, Zhongshan; Wang, Mingbang; Wang, Tao; Wu, Xueli; Jiang, Yi; Cai, Wanshi; Mei, Junpu; Min, Qingjie; Xu, Qiong; Zhou, Bingrui; Guo, Hui; Wang, Ping; Zhou, Wenhao; Hu, Zhengmao; Li, Yingrui; Cai, Tao; Wang, Yi; Xia, Kun; Jiang, Yong-Hui; Sun, Zhong Sheng . Genomic landscapes of Chinese sporadic autism spectrum disorders revealed by whole-genome sequencing. Journal of Genetics and Genomics. 2018. 45: 527-538
Bodian, Dale L.; Vilboux, Thierry; Hourigan, Suchitra K.; Jenevein, Callie L.; Mani, Haresh; Kent, Kathleen C.; Khromykh, Alina; Solomon, Benjamin D.; Hauser, Natalie S. . Genomic analysis of an infant with intractable diarrhea and dilated cardiomyopathy. Molecular Case Studies. 2017. 3: a002055
Whitford, Whitney; Hawkins, Isobel; Glamuzina, Emma; Wilson, Francessa; Marshall, Andrew; Ashton, Fern; Love, Donald R.; Taylor, Juliet; Hill, Rosamund; Lehnert, Klaus; Snell, Russell G.; Jacobsen, Jessie C. . Compound heterozygous SLC19A3 mutations further refine the critical promoter region for biotin-thiamine-responsive basal ganglia disease. Molecular Case Studies. 2017. 3: a001909
Flønes, Irene; Sztromwasser, Paweł; Haugarvoll, Kristoffer; Dölle, Christian; Lykouri, Maria; Schwarzlmüller, Thomas; Jonassen, Inge; Miletic, Hrvoje; Johansson, Stefan; Knappskog, Per M.; Bindoff, Laurence A.; Tzoulis, Charalampos . Novel SLC19A3 Promoter Deletion and Allelic Silencing in Biotin-Thiamine-Responsive Basal Ganglia Encephalopathy. PLOS ONE. 2016. 11: e0149055

Khromykh, Alina; Solomon, Benjamin D; Bodian, Dale L; Leon, Eyby L; Iyer, Ramaswamy K; Baker, Robin L; Ascher, David P; Baveja, Rajiv; Vockley, Joseph G; Niederhuber, John E . Diagnosis of D-Bifunctional Protein Deficiency through Whole-Genome Sequencing: Implications for Cost-Effective Care. Molecular Syndromology. 2015. 6: 141-146
Martin, Hilary C.; Kim, Grace E.; Pagnamenta, Alistair T.; Murakami, Yoshiko; Carvill, Gemma L.; Meyer, Esther; Copley, Richard R.; Rimmer, Andrew; Barcia, Giulia; Fleming, Matthew R.; Kronengold, Jack; Brown, Maile R.; Hudspith, Karl A.; Broxholme, John; Kanapin, Alexander; Cazier, Jean-Baptiste; Kinoshita, Taroh; Nabbout, Rima; ; Bentley, David; McVean, Gil; Heavin, Sinéad; Zaiwalla, Zenobia; McShane, Tony; Mefford, Heather C.; Shears, Deborah; Stewart, Helen; Kurian, Manju A.; Scheffer, Ingrid E.; Blair, Edward; Donnelly, Peter; Kaczmarek, Leonard K.; Taylor, Jenny C. . Clinical whole-genome sequencing in severe early-onset epilepsy reveals new genes and improves molecular diagnosis. Human Molecular Genetics. 2014. 23: 3200-3211
Saunders, Carol Jean; Miller, Neil Andrew; Soden, Sarah Elizabeth; Dinwiddie, Darrell Lee; Noll, Aaron; Abu Alnadi, Noor; Andraws, Nevene; Patterson, Melanie LeAnn; Krivohlavek, Lisa Ann; Fellis, Joel; Humphray, Sean; Saffrey, Peter; Kingsbury, Zoya; Weir, Jacqueline Claire; Betley, Jason; Grocock, Russell James; Margulies, Elliott Harrison; Farrow, Emily Gwendolyn; Artman, Michael; Safina, Nicole Pauline; Petrikin, Joshua Erin; Hall, Kevin Peter; Kingsmore, Stephen Francis . Rapid Whole-Genome Sequencing for Genetic Disease Diagnosis in Neonatal Intensive Care Units. Science Translational Medicine. 2012. 4: 154ra135-154ra135

Bainbridge, Matthew N.; Wiszniewski, Wojciech; Murdock, David R.; Friedman, Jennifer; Gonzaga-Jauregui, Claudia; Newsham, Irene; Reid, Jeffrey G.; Fink, John K.; Morgan, Margaret B.; Gingras, Marie-Claude; Muzny, Donna M.; Hoang, Linh D.; Yousaf, Shahed; Lupski, James R.; Gibbs, Richard A. . Whole-Genome Sequencing for Optimized Patient Management. Science Translational Medicine. 2011. 3: 87re3-87re3
H Rafehi, DJ Szmulewicz, K Pope, M Wallis, J Christodoulou, SM White, MB Delatycki, PJ Lockhart, M Bahlo . Rapid Diagnosis of Spinocerebellar Ataxia 36 in a Three-Generation Family Using Short-Read Whole-Genome Sequencing Data.. Movement disorders : official journal of the Movement Disorder Society . 2020 . 35:
QY Sun, Q Xu, Y Tian, ZM Hu, LX Qin, JX Yang, W Huang, J Xue, JC Li, S Zeng, Y Wang, HX Min, XY Chen, JP Wang, B Xie, F Liang, HN Zhang, CY Wang, LF Lei, XX Yan, HW Xu, RH Duan, K Xia, JY Liu, H Jiang, L Shen, JF Guo, BS Tang . Expansion of GGC repeat in the human-specific NOTCH2NLC gene is associated with essential tremor.. Brain : a journal of neurology . 2020 . 143:
J Chen, JS Wu, T Mize, M Moreno, M Hamid, F Servin, B Bashy, Z Zhao, P Jia, MT Tsuang, KS Kendler, M Xiong, X Chen . A Frameshift Variant in the CHST9 Gene Identified by Family-Based Whole Genome Sequencing Is Associated with Schizophrenia in Chinese Population.. Scientific reports . 2019 . 9:

S Al-Khawaga, I Mohammed, S Saraswathi, B Haris, R Hasnah, A Saeed, H Almabrazi, N Syed, P Jithesh, A El Awwa, A Khalifa, F AlKhalaf, G Petrovski, EM Abdelalim, K Hussain . The clinical and genetic characteristics of permanent neonatal diabetes (PNDM) in the state of Qatar.. Molecular genetics & genomic medicine . 2019 . 7:
J Deng, M Gu, Y Miao, S Yao, M Zhu, P Fang, X Yu, P Li, Y Su, J Huang, J Zhang, J Yu, F Li, J Bai, W Sun, Y Huang, Y Yuan, D Hong, Z Wang . Long-read sequencing identified repeat expansions in the 5'UTR of the <i>NOTCH2NLC</i> gene from Chinese patients with neuronal intranuclear inclusion disease.. Journal of medical genetics . 2019 . 56:
SE Laurenzano, C McFall, L Nguyen, D Savla, NG Coufal, MS Wright, M Tokita, D Dimmock, SF Kingsmore, RS Newfield . Neonatal diabetes mellitus due to a novel variant in the <i>INS</i> gene.. Cold Spring Harbor molecular case studies . 2019 . 5:
K Chen, M Rao, G Guo, F Duru, L Chen, X Chen, J Song, S Hu . Recessive variants in plakophilin-2 contributes to early-onset arrhythmogenic cardiomyopathy with severe heart failure.. Europace : European pacing, arrhythmias, and cardiac electrophysiology : journal of the working groups on cardiac pacing, arrhythmias, and cardiac cellular electrophysiology of the European Society of Cardiology . 2019 . 21:
AC Thuresson, C Soussi Zander, JJ Zhao, J Halvardson, K Maqbool, E Månsson, E Stenninger, U Holmlund, Y Öhrner, L Feuk . Whole genome sequencing of consanguineous families reveals novel pathogenic variants in intellectual disability.. Clinical genetics . 2019 . 95:

DY Chen, S Chowdhury, L Farnaes, JR Friedman, J Honold, DP Dimmock, OBOTRIJJ Gold . Rapid Diagnosis of KCNQ2-Associated Early Infantile Epileptic Encephalopathy Improved Outcome.. Pediatric neurology . 2018 . 86:
NM Sweeney, SA Nahas, S Chowdhury, MD Campo, MC Jones, DP Dimmock, SF Kingsmore, . The case for early use of rapid whole-genome sequencing in management of critically ill infants: late diagnosis of Coffin-Siris syndrome in an infant with left congenital diaphragmatic hernia, congenital heart disease, and recurrent infections.. Cold Spring Harbor molecular case studies . 2018 . 4:
A Hildreth, K Wigby, S Chowdhury, S Nahas, J Barea, P Ordonez, S Batalov, D Dimmock, S Kingsmore, . Rapid whole-genome sequencing identifies a novel homozygous <i>NPC1</i> variant associated with Niemann-Pick type C1 disease in a 7-week-old male with cholestasis.. Cold Spring Harbor molecular case studies . 2017 . 3:
FR Zahir, JC Mwenifumbo, HE Chun, EL Lim, CDM Van Karnebeek, M Couse, KL Mungall, L Lee, N Makela, L Armstrong, CF Boerkoel, SL Langlois, BM McGillivray, SJM Jones, JM Friedman, MA Marra . Comprehensive whole genome sequence analyses yields novel genetic and structural insights for Intellectual Disability.. BMC genomics . 2017 . 18:
G Arno, S Hull, K Carss, A Dev-Borman, C Chakarova, K Bujakowska, LI van den Born, AG Robson, GE Holder, M Michaelides, FP Cremers, E Pierce, FL Raymond, AT Moore, AR Webster . Reevaluation of the Retinal Dystrophy Due to Recessive Alleles of RGR With the Discovery of a Cis-Acting Mutation in CDHR1.. Investigative ophthalmology & visual science . 2016 . 57:

Owen, Mallory J; Niemi, Anna-Kaisa; Dimmock, David P; Speziale, Mark; Nespeca, Mark; Chau, Kevin K; Van Der Kraan, Luca; Wright, Meredith S; Hansen, Christian; Veeraraghavan, Narayanan; Ding, Yan; Lenberg, Jerica; Chowdhury, Shimul; Hobbs, Charlotte A; Batalov, Sergey; Zhu, Zhanyang; Nahas, Shareef A; Gilmer, Sheldon; Knight, Gail; Lefebvre, Sebastien; Reynders, John; Defay, Thomas; Weir, Jacqueline; Thomson, Vicki S; Fraser, Louise; Lajoie, Bryan R; McPhail, Tim K; Mehtalia, Shyamal S; Kunard, Chris M; Hall, Kevin P; Kingsmore, Stephen F . Rapid Sequencing-Based Diagnosis of Thiamine Metabolism Dysfunction Syndrome. New England Journal of Medicine. 2021. 384: 2159-2161

Supplementary Figure 1. Bubble plot of study size*, diagnostic yield and clinical phenotype.

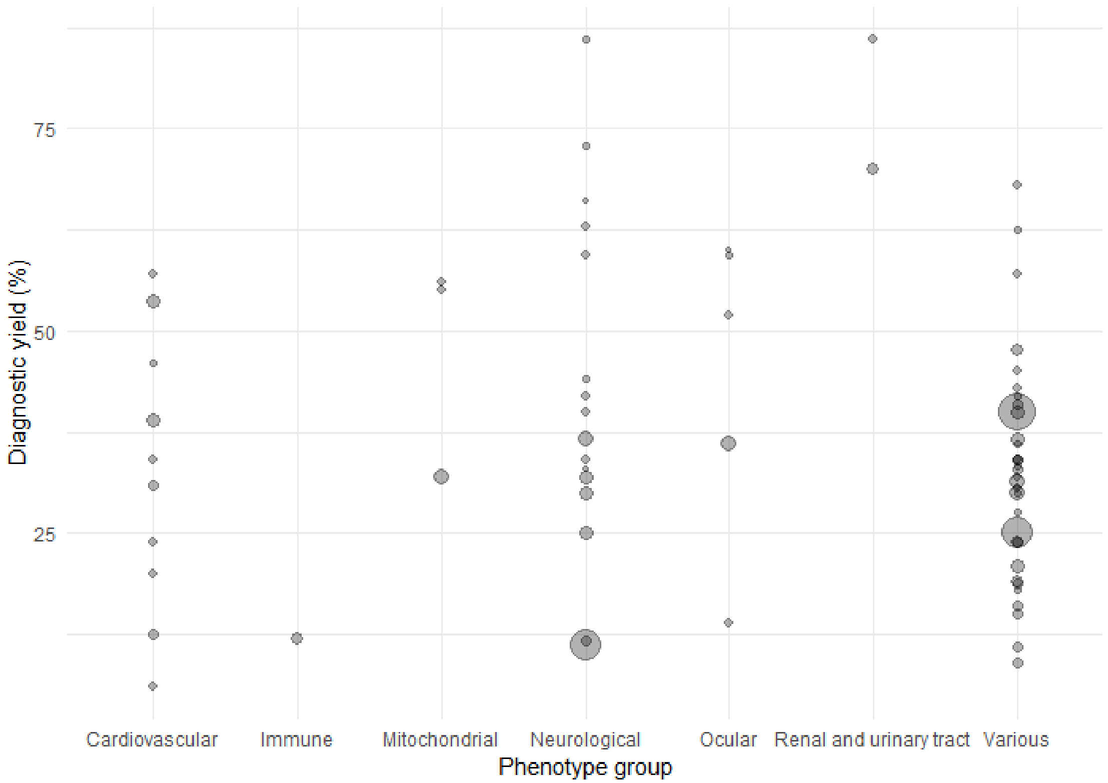

*Study cohort size is denoted by bubble size.

Supplementary Methods

Data Extraction Form

1. Please indicate whether any of the authors are affiliated with a Medical Genome Initiative member institution. Select all that apply.
    a. Baylor Genetics/Baylor College of Medicine
    b. Broad Institute of MIT and Harvard
    c. HudsonAlpha Institute for Biotechnology
    d. Illumina Inc
    e. Mayo Clinic
    f. New York Genome Center
    g. The Hospital for Sick Children (Sick Kids)
    h. Rady Children's Institute for Genomic Medicine
    i. Stanford Medicine
2. Country (write-in)
3. What is the study population?
    a. Pediatric inpatient/acute care
    b. Pediatric outpatient
    c. Adult outpatient
    d. Mixed
4. What is the primary phenotype? (write-in; note if multiple phenotypes)
5. Please indicate the study design.
    a. Prospective

    b. Retrospective
    c. Randomized Clinical Trial (RCT)
    d. Case report
    e. Case series
    f. Meta-analysis

6. Please indicate whether the study was conducted at a single site or across multiple sites.
    a. Single site
    b. Multiple sites
7. Did the study include a comparison group?
    a. Yes
    b. No
8. Did the study include a within-patient comparison? (i.e., patients received both treatments/exposures)
    a. Yes
    b. No
9. Please indicate the number of probands sequenced (write in)
10. Please indicate the family testing structure for the majority (>75%) of the study population
    a. Proband only
    b. Duo (e.g., child-one parent)
    c. Trio (e.g., child-parentS)
    d. Mixed
    e. Other
11. Comment on family testing structure (write in)

12. What was the intervention?
    a. GS
    b. GS plus another test
13. Please indicate the overall diagnostic yield for the primary phenotype (i.e., exclude secondary findings)
14. Use the space below to note the diagnostic yield(s) of any subgroups, other tests, etc.
15. Please indicate the variant types that were indicated as part of the test definition (i.e., what variants were detectable by the test). Select all that apply
    a. SNV
    b. Small (<150 bp) insertions and deletions (indels)
    c. CNV
    d. Runs of homozygosity (ROH)
    e. Non-CNV structural variants (SV)
    f. Variants in regions of high homology or known pseudogenes (e.g., SMN1/SMN2)
    g. Short tandem repeat (STR) expansions
    h. Mitochondrial variants
    i. Mosaic variants
16. Please indicate the variant types that contributed to the molecular diagnoses in the study cohort. Select all that apply.
    a. SNV
    b. Small (<150 bp) insertions and deletions (indels)
    c. CNV
    d. Runs of homozygosity (ROH)
    e. Non-CNV structural variants (SV)

f. Variants in regions of high homology or known pseudogenes (e.g., SMN1/SMN2)
g. Short tandem repeat (STR) expansions
h. Mitochondrial variants
i. Mosaic variants

17. Were secondary findings reported?
    a. Yes
    b. No
18. If ‘Yes’ for question 17, please indicate the variant types that were reported as SF. Select all that apply.
    a. SNV
    b. Small (<150 bp) insertions and deletions (indels)
    c. CNV
    d. Runs of homozygosity (ROH)
    e. Non-CNV structural variants (SV)
    f. Variants in regions of high homology or known pseudogenes (e.g., SMN1/SMN2)
    g. Short tandem repeat (STR) expansions
    h. Mitochondrial variants
    i. Mosaic variants
19. Did the study report measures of clinical utility (e.g., change in management, avoidance of unnecessary procedures)?
    a. Yes
    b. No
20. Clinical utility details
    a. What percent of patients experienced a change in management as a result of a molecular diagnosis?

b. What percent of patients experienced a change in management in non-diagnostic cases?
c. If reported, please indicate the time to result (days)
d. How was follow-up information obtained? (e.g., HER, clinical interview)

21. Diagnostic efficacy: Did the test help the clinician establish a diagnosis? Did the test influence the clinician's decision making about the differential diagnosis they hold for a patient?
    a. Yes
    b. No
    c. N/A
22. If 'Yes' for question 21, please indicate the type or level of evidence
    a. Descriptive (e.g., individual case examples)
    b. Quantitative (e.g., cost analysis, % identified at-risk relatives)
    c. Both
23. Diagnostic efficacy comments (write-in)
24. Therapeutic efficacy: Did the test aid in planning treatment? Did the test change or avert planned treatment?
    a. Yes
    b. No
    c. N/A
25. If 'Yes' for question 24, please indicate the type or level of evidence
    a. Descriptive (e.g., individual case examples)
    b. Quantitative (e.g., cost analysis, % identified at-risk relatives)
    c. Both
26. Therapeutic efficacy comments (write-in)

27. Patient outcome efficacy: Did patients who received the test fare better than similar patients who did not?
    a. Yes
    b. No
    c. N/A
28. If ‘Yes’ for question 27, please indicate the type or level of evidence
    a. Descriptive (e.g., individual case examples)
    b. Quantitative (e.g., cost analysis, % identified at-risk relatives)
    c. Both
29. Patient outcome efficacy comments (write-in)
30. Societal efficacy: Is the test cost-effective? Is it acceptable to society?
    a. Yes
    b. No
    c. N/A
31. If ‘Yes’ for question 30, please indicate the type or level of evidence
    a. Descriptive (e.g., individual case examples)
    b. Quantitative (e.g., cost analysis, % identified at-risk relatives)
    c. Both
32. Societal efficacy comments

Study quality assessment (adapted from the American College of Radiology Appropriateness Criteria)

1. Study type
    a. Diagnostic study
    b. Therapeutic study

2. Statistical measure: Does the study include a statistical measure of results (e.g., calculated mean)?
    a. Yes
    b. No
3. Statistical measure comments
4. Uncertainty measure: Does the study include appropriate uncertainty measure(s)? Examples: standard errors, confidence intervals, p-values, statistical comparison test such as t-test.
    a. Yes
    b. No
5. Uncertainty measure comments
6. Prospective: Was the study designed prior to data collection?
    a. Yes
    b. No
7. Prospective comments
8. Systematic recruitment: Did the study design minimize selection bias through systematic recruitment?
    a. Yes
    b. No
9. Systematic recruitment comments
10. Consecutive series: Did the study design minimize selection bias through consecutive series?
    a. Yes
    b. No
11. Consecutive series comments
12. Standard of reference: Did the study identify a standard of reference?

   a. Gold standard or equivalent
   b. Standard set by comparison between two or more tests
   c. No standard of reference
13. Standard of reference comments
14. Reference standard applied: Did the study consistently compare the standard of reference to the index standard?
   a. Yes
   b. No
15. Reference standard applied comments
16. Clinical correlation: Did the study provide a comparison between the molecular test result and the clinical phenotype?
   a. Yes
   b. No
17. Clinical correlation comments
18. Index test results: Were index test results interpreted without knowledge of the reference standard results? Were comparable protections in place?
   a. Yes
   b. No
19. Index test results comments
20. Reference standard results: Were referenced test results interpreted without knowledge of the index standard results? Were comparable protections in place?
   a. Yes
   b. No
21. Reference standard results comments

22. Study quality (calculated based on responses to questions 1,2,4,6,8,10,12,14,16,18 and 20)

a. Category 1
b. Category 2
c. Category 3
d. Category 4

Supplementary Methods

Study quality components[1]

1. Uncertainty measure included in statistical analysis of results
2. Prospective: The study was designed prior to data collection
3. Systematic recruitment or consecutive series to reduce selection bias
4. Standard of reference, preferable the gold standard, clearly defined
5. Reference standard applied consistently to all subjects
6. At least two independent readers to reduce bias introduced by the ability of the readers of the diagnostic tests
7. Index test results blinded from reference standard
8. Reference standard results blinded from index results

[1] Kurth, D. A., Karmazyn, B. K., Waldrip, C. A., Chatfield, M. & Lockhart, M. E. ACR Appropriateness Criteria; Methodology. *Journal of the American College of Radiology* **18**, S240-S250 (2021).

Supplementary Results. Results of ratings rounds used during consensus development. Percentages reflect how panel members voted for each round.

Recommendation 1

	1 strongly disagree	2 disagree	3 Moderately disagree	4 Slightly disagree	5 Undecided	6 Slightly agree	7 Moderately agree	8 agree	9 Strongly agree
Round 1						8.3%	8.3%	33.3%	50%
Round 2					12.5%			50%	37.5%
Round 3						9.1%	9.1%	27.3%	54.5%
Round 4								25%	75%

Recommendation 2

	1 strongly disagree	2 disagree	3 Moderately disagree	4 Slightly disagree	5 Undecided	6 Slightly agree	7 Moderately agree	8 agree	9 Strongly agree
Round 1					16.7%		25%	25%	25%
Round 2							25%	37.5%	37.5%
Round 3								20%	80%

Recommendation 3

	1 strongly disagree	2 disagree	3 Moderately disagree	4 Slightly disagree	5 Undecided	6 Slightly agree	7 Moderately agree	8 agree	9 Strongly agree
Round 1					25%	8.3%	16.7%	33.3%	8.3%
Round 2							62.5%	25%	12.5%
Round 3							9.1%	18.2%	72.7%
Round 4								20%	80%

Recommendation 4

	1 strongly disagree	2 disagree	3 Moderately disagree	4 Slightly disagree	5 Undecided	6 Slightly agree	7 Moderately agree	8 agree	9 Strongly agree
Round 1					8.3%			8.3%	75%
Round 2							18.2%	9.1%	72.7%
Round 3								25%	75%

Recommendation 5

	1 strongly disagree	2 disagree	3 Moderately disagree	4 Slightly disagree	5 Undecided	6 Slightly agree	7 Moderately agree	8 agree	9 Strongly agree
Round 1							9.1%	18.2%	72.7%
Round 2								18.2%	81.8%

Supplementary Tables I-IV References

1 Group, T. N. S. Effect of Whole-Genome Sequencing on the Clinical Management of Acutely Ill Infants With Suspected Genetic Disease: A Randomized Clinical Trial. *JAMA Pediatrics* (2021). https://doi.org:10.1001/jamapediatrics.2021.3496

2 Maron, J. L. *et al.* Novel Variant Findings and Challenges Associated With the Clinical Integration of Genomic Testing: An Interim Report of the Genomic Medicine for Ill Neonates and Infants (GEMINI) Study. *JAMA Pediatr* **175**, e205906 (2021). https://doi.org:10.1001/jamapediatrics.2020.5906

3 Kingsmore, S. F. *et al.* A Randomized, Controlled Trial of the Analytic and Diagnostic Performance of Singleton and Trio, Rapid Genome and Exome Sequencing in Ill Infants. *Am J Hum Genet* **105**, 719-733 (2019). https://doi.org:10.1016/j.ajhg.2019.08.009

4 Dimmock, D. P. *et al.* An RCT of Rapid Genomic Sequencing among Seriously Ill Infants Results in High Clinical Utility, Changes in Management, and Low Perceived Harm. *Am J Hum Genet* **107**, 942-952 (2020). https://doi.org:10.1016/j.ajhg.2020.10.003

5 Cakici, J. A. *et al.* A Prospective Study of Parental Perceptions of Rapid Whole-Genome and -Exome Sequencing among Seriously Ill Infants. *Am J Hum Genet* **107**, 953-962 (2020). https://doi.org:10.1016/j.ajhg.2020.10.004

6 Petrikin, J. E. *et al.* The NSIGHT1-randomized controlled trial: rapid whole-genome sequencing for accelerated etiologic diagnosis in critically ill infants. *NPJ Genom Med* **3**, 6 (2018). https://doi.org:10.1038/s41525-018-0045-8

7 Berrios, C., Koertje, C., Noel-MacDonnell, J., Soden, S. & Lantos, J. Parents of newborns in the NICU enrolled in genome sequencing research: hopeful, but not naive. *Genet Med* (2019). https://doi.org:10.1038/s41436-019-0644-5

8 Bowling, K. M. *et al.* Genome sequencing as a first-line diagnostic test for hospitalized infants. *Genet Med* **24**, 851-861 (2022). https://doi.org:10.1016/j.gim.2021.11.020

9 Dimmock, D. *et al.* Project Baby Bear: Rapid precision care incorporating rWGS in 5 California children's hospitals demonstrates improved clinical outcomes and reduced costs of care. *Am J Hum Genet* **108**, 1231-1238 (2021). https://doi.org:10.1016/j.ajhg.2021.05.008

10 Sweeney, N. M. *et al.* Rapid whole genome sequencing impacts care and resource utilization in infants with congenital heart disease. *NPJ Genom Med* **6**, 29 (2021). https://doi.org:10.1038/s41525-021-00192-x

11 Wang, H. *et al.* Optimized trio genome sequencing (OTGS) as a first-tier genetic test in critically ill infants: practice in China. *Hum Genet* **139**, 473-482 (2020). https://doi.org:10.1007/s00439-019-02103-8

12 Sanford, E. F. *et al.* Rapid Whole Genome Sequencing Has Clinical Utility in Children in the PICU. *Pediatr Crit Care Med* (2019). https://doi.org:10.1097/pcc.0000000000002056

13 Hauser, N. S. *et al.* Experience with genomic sequencing in pediatric patients with congenital cardiac defects in a large community hospital. *Mol Genet Genomic Med* **6**, 200-212 (2018). https://doi.org:10.1002/mgg3.357

14 Mestek-Boukhibar, L. *et al.* Rapid Paediatric Sequencing (RaPS): comprehensive real-life workflow for rapid diagnosis of critically ill children. *J Med Genet* (2018). https://doi.org:10.1136/jmedgenet-2018-105396

15 van Diemen, C. C. *et al.* Rapid Targeted Genomics in Critically Ill Newborns. *Pediatrics* **140** (2017). https://doi.org:10.1542/peds.2016-2854

16 Wu, B. *et al.* Application of Full-Spectrum Rapid Clinical Genome Sequencing Improves Diagnostic Rate and Clinical Outcomes in Critically Ill Infants in the China Neonatal Genomes Project. *Crit Care Med* (2021). https://doi.org:10.1097/ccm.0000000000005052

17 Palmquist, R. *et al.* Evaluating use of changing technologies for rapid next-generation sequencing in pediatrics. *Pediatr Res* (2022). https://doi.org:10.1038/s41390-022-01965-5

18 Farnaes, L. *et al.* Rapid whole-genome sequencing decreases infant morbidity and cost of hospitalization. *NPJ Genom Med* **3**, 10 (2018). https://doi.org:10.1038/s41525-018-0049-4

19 Willig, L. K. *et al.* Whole-genome sequencing for identification of Mendelian disorders in critically ill infants: a retrospective analysis of diagnostic and clinical findings. *Lancet Respir Med* **3**, 377-387 (2015). https://doi.org:10.1016/s2213-2600(15)00139-3

20 Palmer, E. E. *et al.* Diagnostic Yield of Whole Genome Sequencing After Nondiagnostic Exome Sequencing or Gene Panel in Developmental and Epileptic Encephalopathies. *Neurology* **96**, e1770-e1782 (2021). https://doi.org:10.1212/wnl.0000000000011655

21 Zou, D. *et al.* Genome sequencing of 320 Chinese children with epilepsy: a clinical and molecular study. *Brain* **144**, 3623-3634 (2021). https://doi.org:10.1093/brain/awab233

22 Al-Hassnan, Z. N. *et al.* Categorized Genetic Analysis in Childhood-Onset Cardiomyopathy. *Circ Genom Precis Med* **13**, 504-514 (2020). https://doi.org:10.1161/circgen.120.002969

23 Costain, G. *et al.* Genome Sequencing as a Diagnostic Test in Children With Unexplained Medical Complexity. *JAMA Netw Open* **3**, e2018109 (2020). https://doi.org:10.1001/jamanetworkopen.2020.18109

24 Jackson, D. *et al.* Molecular diagnostic challenges for non-retinal developmental eye disorders in the United Kingdom. *Am J Med Genet C Semin Med Genet* **184**, 578-589 (2020). https://doi.org:10.1002/ajmg.c.31837

25 Reuter, M. S. *et al.* The Cardiac Genome Clinic: implementing genome sequencing in pediatric heart disease. *Genetics in Medicine* **22**, 1015-1024 (2020). https://doi.org:10.1038/s41436-020-0757-x

26 Riley, L. G. *et al.* The diagnostic utility of genome sequencing in a pediatric cohort with suspected mitochondrial disease. *Genetics in Medicine* **22**, 1254-1261 (2020). https://doi.org:10.1038/s41436-020-0793-6

27 Scocchia, A. *et al.* Clinical whole genome sequencing as a first-tier test at a resource-limited dysmorphology clinic in Mexico. *NPJ Genom Med* **4**, 5 (2019). https://doi.org:10.1038/s41525-018-0076-1

28 Hull, S. *et al.* Clinical and Molecular Characterization of Familial Exudative Vitreoretinopathy Associated With Microcephaly. *Am J Ophthalmol* **207**, 87-98 (2019). https://doi.org:10.1016/j.ajo.2019.05.001

29 Thiffault, I. *et al.* Clinical genome sequencing in an unbiased pediatric cohort. *Genet Med* **21**, 303-310 (2019). https://doi.org:10.1038/s41436-018-0075-8

30 Alankarage, D. *et al.* Identification of clinically actionable variants from genome sequencing of families with congenital heart disease. *Genetics in Medicine* **21**, 1111-1120 (2019). https://doi.org:10.1038/s41436-018-0296-x

31 Ostrander, B. E. P. *et al.* Whole-genome analysis for effective clinical diagnosis and gene discovery in early infantile epileptic encephalopathy. *NPJ Genom Med* **3**, 22 (2018). https://doi.org:10.1038/s41525-018-0061-8

32 Costain, G. *et al.* Periodic reanalysis of whole-genome sequencing data enhances the diagnostic advantage over standard clinical genetic testing. *Eur J Hum Genet* **26**, 740-744 (2018). https://doi.org:10.1038/s41431-018-0114-6
33 Lionel, A. C. *et al.* Improved diagnostic yield compared with targeted gene sequencing panels suggests a role for whole-genome sequencing as a first-tier genetic test. *Genet Med* (2017). https://doi.org:10.1038/gim.2017.119
34 Bowling, K. M. *et al.* Genomic diagnosis for children with intellectual disability and/or developmental delay. *Genome Med* **9**, 43 (2017). https://doi.org:10.1186/s13073-017-0433-1
35 Yuen, R. *et al.* Whole genome sequencing resource identifies 18 new candidate genes for autism spectrum disorder. *Nat Neurosci* **20**, 602-611 (2017). https://doi.org:10.1038/nn.4524
36 Bick, D. *et al.* Successful Application of Whole Genome Sequencing in a Medical Genetics Clinic. *J Pediatr Genet* **6**, 61-76 (2017). https://doi.org:10.1055/s-0036-1593968
37 Stavropoulos, D. J. *et al.* Whole Genome Sequencing Expands Diagnostic Utility and Improves Clinical Management in Pediatric Medicine. *NPJ Genom Med* **1** (2016). https://doi.org:10.1038/npjgenmed.2015.12
38 Lesurf, R. *et al.* Whole genome sequencing delineates regulatory, copy number, and cryptic splice variants in early onset cardiomyopathy. *NPJ Genom Med* **7**, 18 (2022). https://doi.org:10.1038/s41525-022-00288-y
39 Miller, K. A. *et al.* Diagnostic value of exome and whole genome sequencing in craniosynostosis. *J Med Genet* **54**, 260-268 (2017). https://doi.org:10.1136/jmedgenet-2016-104215
40 Brockman, D. G. *et al.* Randomized prospective evaluation of genome sequencing versus standard-of-care as a first molecular diagnostic test. *Genet Med* (2021). https://doi.org:10.1038/s41436-021-01193-y
41 Vanderver, A. *et al.* Randomized Clinical Trial of First-Line Genome Sequencing in Pediatric White Matter Disorders. *Ann Neurol* (2020). https://doi.org:10.1002/ana.25757
42 Ewans, L. J. *et al.* Whole exome and genome sequencing in mendelian disorders: a diagnostic and health economic analysis. *Eur J Hum Genet* (2022). https://doi.org:10.1038/s41431-022-01162-2
43 Bhatia, N. S. *et al.* Singapore Undiagnosed Disease Program: Genomic Analysis aids Diagnosis and Clinical Management. *Arch Dis Child* **106**, 31-37 (2021). https://doi.org:10.1136/archdischild-2020-319180
44 Chan, H. W. *et al.* Prospective Study of the Phenotypic and Mutational Spectrum of Ocular Albinism and Oculocutaneous Albinism. *Genes (Basel)* **12** (2021). https://doi.org:10.3390/genes12040508
45 Stranneheim, H. *et al.* Integration of whole genome sequencing into a healthcare setting: high diagnostic rates across multiple clinical entities in 3219 rare disease patients. *Genome Med* **13**, 40 (2021). https://doi.org:10.1186/s13073-021-00855-5
46 Mallawaarachchi, A. C. *et al.* Genomic diagnostics in polycystic kidney disease: an assessment of real-world use of whole-genome sequencing. *Eur J Hum Genet* **29**, 760-770 (2021). https://doi.org:10.1038/s41431-020-00796-4
47 Schon, K. R. *et al.* Use of whole genome sequencing to determine genetic basis of suspected mitochondrial disorders: cohort study. *Bmj* **375**, e066288 (2021). https://doi.org:10.1136/bmj-2021-066288
48 Dahl, S. *et al.* Whole genome sequencing unveils genetic heterogeneity in optic nerve hypoplasia. *PLoS One* **15**, e0228622 (2020). https://doi.org:10.1371/journal.pone.0228622

49 French, C. E. *et al.* Whole genome sequencing reveals that genetic conditions are frequent in intensively ill children. *Intensive Care Med* **45**, 627-636 (2019). https://doi.org:10.1007/s00134-019-05552-x

50 Kumar, K. R. *et al.* Whole genome sequencing for the genetic diagnosis of heterogenous dystonia phenotypes. *Parkinsonism Relat Disord* **69**, 111-118 (2019). https://doi.org:10.1016/j.parkreldis.2019.11.004

51 Liu, H. Y. *et al.* Diagnostic and clinical utility of whole genome sequencing in a cohort of undiagnosed Chinese families with rare diseases. *Sci Rep* **9**, 19365 (2019). https://doi.org:10.1038/s41598-019-55832-1

52 Lee, H. F., Chi, C. S. & Tsai, C. R. Diagnostic yield and treatment impact of whole-genome sequencing in paediatric neurological disorders. *Dev Med Child Neurol* (2020). https://doi.org:10.1111/dmcn.14722

53 Schluth-Bolard, C. *et al.* Whole genome paired-end sequencing elucidates functional and phenotypic consequences of balanced chromosomal rearrangement in patients with developmental disorders. *J Med Genet* **56**, 526-535 (2019). https://doi.org:10.1136/jmedgenet-2018-105778

54 Shashi, V. *et al.* A comprehensive iterative approach is highly effective in diagnosing individuals who are exome negative. *Genet Med* **21**, 161-172 (2019). https://doi.org:10.1038/s41436-018-0044-2

55 Splinter, K. *et al.* Effect of Genetic Diagnosis on Patients with Previously Undiagnosed Disease. *N Engl J Med* (2018). https://doi.org:10.1056/NEJMoa1714458

56 Bagnall, R. D. *et al.* Whole Genome Sequencing Improves Outcomes of Genetic Testing in Patients With Hypertrophic Cardiomyopathy. *Journal of the American College of Cardiology* **72**, 419-429 (2018). https://doi.org:https://doi.org/10.1016/j.jacc.2018.04.078

57 Alfares, A. *et al.* Whole-genome sequencing offers additional but limited clinical utility compared with reanalysis of whole-exome sequencing. *Genet Med* (2018). https://doi.org:10.1038/gim.2018.41

58 Hamdan, F. F. *et al.* High Rate of Recurrent De Novo Mutations in Developmental and Epileptic Encephalopathies. *Am J Hum Genet* **101**, 664-685 (2017). https://doi.org:10.1016/j.ajhg.2017.09.008

59 Kumar, K. R. *et al.* Defining the genetic basis of early onset hereditary spastic paraplegia using whole genome sequencing. *Neurogenetics* **17**, 265-270 (2016). https://doi.org:10.1007/s10048-016-0495-z

60 Bloss, C. S. *et al.* A genome sequencing program for novel undiagnosed diseases. *Genet Med* **17**, 995-1001 (2015). https://doi.org:10.1038/gim.2015.21

61 Gilissen, C. *et al.* Genome sequencing identifies major causes of severe intellectual disability. *Nature* **511**, 344-347 (2014). https://doi.org:10.1038/nature13394

62 Lindstrand, A. *et al.* Genome sequencing is a sensitive first-line test to diagnose individuals with intellectual disability. *Genet Med* **24**, 2296-2307 (2022). https://doi.org:10.1016/j.gim.2022.07.022

63 Naess, K. *et al.* Clinical Presentation, Genetic Etiology, and Coenzyme Q10 Levels in 55 Children with Combined Enzyme Deficiencies of the Mitochondrial Respiratory Chain. *J Pediatr* **228**, 240-251.e242 (2021). https://doi.org:10.1016/j.jpeds.2020.08.025

64 Smedley, D. *et al.* 100,000 Genomes Pilot on Rare-Disease Diagnosis in Health Care - Preliminary Report. *N Engl J Med* **385**, 1868-1880 (2021). https://doi.org:10.1056/NEJMoa2035790

65 Helman, G. *et al.* Genome sequencing in persistently unsolved white matter disorders. *Ann Clin Transl Neurol* **7**, 144-152 (2020). https://doi.org:10.1002/acn3.50957
66 Gross, A. M. *et al.* Copy-number variants in clinical genome sequencing: deployment and interpretation for rare and undiagnosed disease. *Genet Med* **21**, 1121-1130(2019).
67 Soden, S. E. *et al.* Effectiveness of exome and genome sequencing guided by acuity of illness for diagnosis of neurodevelopmental disorders. *Sci Transl Med* **6**, 265ra168 (2014). https://doi.org:10.1126/scitranslmed.3010076
68 Cirino, A. L. *et al.* A Comparison of Whole Genome Sequencing to Multigene Panel Testing in Hypertrophic Cardiomyopathy Patients. *Circ Cardiovasc Genet* **10** (2017). https://doi.org:10.1161/circgenetics.117.001768
69 Shoemark, A. *et al.* Genome sequencing reveals underdiagnosis of primary ciliary dyskinesia in bronchiectasis. *Eur Respir J* **60** (2022). https://doi.org:10.1183/13993003.00176-2022
70 Aryan, Z. *et al.* Moving Genomics to Routine Care: An Initial Pilot in Acute Cardiovascular Disease. *Circ Genom Precis Med* **13**, 406-416 (2020). https://doi.org:10.1161/circgen.120.002961
71 Minoche, A. E. *et al.* Genome sequencing as a first-line genetic test in familial dilated cardiomyopathy. *Genetics in medicine : official journal of the American College of Medical Genetics* **21**, 650-662 (2019). https://doi.org:10.1038/s41436-018-0084-7
72 Ellingford, J. M. *et al.* Whole Genome Sequencing Increases Molecular Diagnostic Yield Compared with Current Diagnostic Testing for Inherited Retinal Disease. *Ophthalmology* **123**, 1143-1150 (2016). https://doi.org:10.1016/j.ophtha.2016.01.009
73 Mallawaarachchi, A. C. *et al.* Whole-genome sequencing overcomes pseudogene homology to diagnose autosomal dominant polycystic kidney disease. *Eur J Hum Genet* **24**, 1584-1590 (2016). https://doi.org:10.1038/ejhg.2016.48
74 Kang, C. *et al.* High Degree of Genetic Heterogeneity for Hereditary Cerebellar Ataxias in Australia. *Cerebellum* **18**, 137-146 (2019). https://doi.org:10.1007/s12311-018-0969-7
