# Supplementary material for: Evidence review and considerations for use of first line genome sequencing to diagnose rare genetic disorders
Source: NPJ Genom Med. 2024 Feb 26;9:15. doi: 10.1038/s41525-024-00396-x (PMC10897481; doi:10.1038/s41525-024-00396-x)
Supplement: Supplementary file 2 — REPORTING SUMMARY [file 41525_2024_396_MOESM2_ESM.pdf]

nature portfolio

Corresponding author(s): Dr Kristen Wigby

Last updated by author(s): Oct 26, 2023

Reporting Summary

Nature Portfolio wishes to improve the reproducibility of the work that we publish. This form provides structure for consistency and transparency in reporting. For further information on Nature Portfolio policies, see our Editorial Policies and the Editorial Policy Checklist.

Statistics

For all statistical analyses, confirm that the following items are present in the figure legend, table legend, main text, or Methods section.

n/a	Confirmed	
☐	☒	The exact sample size (*n*) for each experimental group/condition, given as a discrete number and unit of measurement
☐	☒	A statement on whether measurements were taken from distinct samples or whether the same sample was measured repeatedly
☐	☒	The statistical test(s) used AND whether they are one- or two-sided<br>*Only common tests should be described solely by name; describe more complex techniques in the Methods section.*
☒	☐	A description of all covariates tested
☐	☒	A description of any assumptions or corrections, such as tests of normality and adjustment for multiple comparisons
☐	☒	A full description of the statistical parameters including central tendency (e.g. means) or other basic estimates (e.g. regression coefficient) AND variation (e.g. standard deviation) or associated estimates of uncertainty (e.g. confidence intervals)
☐	☒	For null hypothesis testing, the test statistic (e.g. *F*, *t*, *r*) with confidence intervals, effect sizes, degrees of freedom and *P* value noted<br>*Give P values as exact values whenever suitable.*
☒	☐	For Bayesian analysis, information on the choice of priors and Markov chain Monte Carlo settings
☒	☐	For hierarchical and complex designs, identification of the appropriate level for tests and full reporting of outcomes
☒	☐	Estimates of effect sizes (e.g. Cohen's *d*, Pearson's *r*), indicating how they were calculated

Our web collection on statistics for biologists contains articles on many of the points above.

Software and code

Policy information about availability of computer code

Data collection	Each reference that moved on to the full text review and data extraction phases was reviewed by two independent reviewers. Custom data extraction forms were created in DistillerSR to collect a broad range of information related to study characteristics (e.g., study setting and patient cohort) and outcome data (e.g., diagnostic yield). A detailed list of information gathered during initial data extraction can be found in the Supplementary Methods – Data Extraction Form.
Data analysis	The data was exported from Distiller SR to Microsoft Excel to generate descriptive statistics and summary tables and figures. An exploratory meta-analysis using a random effects model evaluated diagnostic yield based on cohort size and diagnosed cases per cohort. All calculations were performed in R using the meta package.

For manuscripts utilizing custom algorithms or software that are central to the research but not yet described in published literature, software must be made available to editors and reviewers. We strongly encourage code deposition in a community repository (e.g. GitHub). See the Nature Portfolio guidelines for submitting code & software for further information.

Data

Policy information about availability of data

All manuscripts must include a data availability statement. This statement should provide the following information, where applicable:

- Accession codes, unique identifiers, or web links for publicly available datasets
- A description of any restrictions on data availability
- For clinical datasets or third party data, please ensure that the statement adheres to our policy

The datasets generated and/or analyzed during the current study are available in the GitHub repository, https://github.com/MedicalGenomeInitiative/Patient-Selection.

Research involving human participants, their data, or biological material

Policy information about studies with human participants or human data. See also policy information about sex, gender (identity/presentation), and sexual orientation and race, ethnicity and racism.

Reporting on sex and gender	We did not not include data on sex of participants from individual cohort studies reviewed during this descriptive literature review and analysis.
Reporting on race, ethnicity, or other socially relevant groupings	We did not not include data on race and ethnicity of participants from individual cohort studies reviewed during this descriptive literature review and analysis. We did report on countries where studies were conducted.
Population characteristics	This literature analysis included 71 studies (observational cohort study, clinical trial, or retrospective analysis) of whole genome sequencing on patient populations spanning neonatal, pediatric, and adult cohorts in different clinical settings (ambulatory, hospitalized, and mixed) were including comprising over 13,000 individuals who receive whole genome sequencing as a diagnostic study for suspected rare genetic disorders.
Recruitment	No primary recruitment was conducted for this literature review.
Ethics oversight	Not indicated for literature review and analysis.

Note that full information on the approval of the study protocol must also be provided in the manuscript.

Field-specific reporting

Please select the one below that is the best fit for your research. If you are not sure, read the appropriate sections before making your selection.

☒ Life sciences ☐ Behavioural & social sciences ☐ Ecological, evolutionary & environmental sciences

For a reference copy of the document with all sections, see nature.com/documents/nr-reporting-summary-flat.pdf

Life sciences study design

All studies must disclose on these points even when the disclosure is negative.

Sample size	Sample size was determined by the number of studies meeting inclusion criteria during the period of literature search. The searches were initially performed on 12 March 2020 and updated on 01 August 2022. Studies published between 1 January 2011 to 01 August 2022 were included. We comment on the limitations of the number of studies and individual cohort sizes in the discussion and the limitations of the analysis.
Data exclusions	Inclusion and exclusion criteria for the literature search were prespecified. All studies meeting inclusion criteria were analysed. The following exclusion criteria were applied: lack of assessment of diagnostic yield or clinical utility, inappropriate publication type (e.g., reviews, editorials, conference abstracts), and inappropriate study design (e.g., animal studies, WES-only studies, case reports). Case reports and case series represent valuable and important pieces of evidence, and although they were technically excluded from the review, a separate repository was created to keep track of them
Replication	Each reference that moved on to the full text review and data extraction phases was reviewed by two independent reviewers. Further data extraction was subsequently conducted to obtain additional details on GS an analysis
Randomization	none
Blinding	none

Reporting for specific materials, systems and methods

We require information from authors about some types of materials, experimental systems and methods used in many studies. Here, indicate whether each material, system or method listed is relevant to your study. If you are not sure if a list item applies to your research, read the appropriate section before selecting a response.

Materials & experimental systems

n/a	Involved in the study
☒	☐ Antibodies
☒	☐ Eukaryotic cell lines
☒	☐ Palaeontology and archaeology
☒	☐ Animals and other organisms
☒	☐ Clinical data
☒	☐ Dual use research of concern
☒	☐ Plants

Methods

n/a	Involved in the study
☒	☐ ChIP-seq
☒	☐ Flow cytometry
☒	☐ MRI-based neuroimaging

Plants

Seed stocks	n/a
Novel plant genotypes	n/a
Authentication	n/a
